# Influence of land-use history and ENSO on the flora of the Southern Line Islands

**DOI:** 10.1371/journal.pone.0341582

**Published:** 2026-02-06

**Authors:** Lauren Nerfa, J. Michael Fay, Allyson Earl, Alan M. Friedlander, Enric Sala, Tamara Ticktin

**Affiliations:** 1 School of Life Sciences, University of Hawaiʻi at Mānoa, Hawaiʻi, United States of America; 2 National Geographic Society, Washington, D.C., United States of America; 3 University of Hawaiʻi Cooperative Extension, Hawaiʻi, United States of America; 4 Hawaiʻi Institute of Marine Biology, University of Hawaiʻi at Mānoa, Hawaiʻi, United States of America; National Cheng Kung University, TAIWAN

## Abstract

Remote tropical islands host unique ecosystems with rare species that have been historically affected by habitat degradation and species introductions, and now by climate change. However, we know little about the current ecological conditions of remote island ecosystems, particularly after the abandonment of commercial land uses. The Southern Line Islands, Kiribati, are among the least studied island groups in the Pacific. These islands have a history of land use, including guano extraction and coconut plantations (1800s to early 1900s), but have no current human uses or habitation. They have been exposed to El Niño Southern Oscillation (ENSO) events, particularly the strong 2015−2016 event. We studied the vegetation of Flint Island, Millennium Atoll, and Vostok Island to assess vegetation succession since the cessation of land-use, and whether they have been impacted by ENSO. Specifically, we drew on field surveys and satellite images from 2009 and 2021, and assessed changes in floristic characteristics between (i) current and historic surveys; (ii) islands with different land-use histories; and (iii) before and after the 2015−2016 ENSO. We found that extant species richness differed from the historic studies, due in large part to the disappearance of some non-native species on Millennium and Flint, and the emergence of some previously undocumented native species across the islands. Species composition differed across islands; Vostok Island has few species compared to Flint or Millennium due to its diminutive size, and remains dominated by *Pisonia* forest. There were few differences in plant species frequency and composition for the islands between 2009 and 2021, but normalized difference vegetation index (NDVI) values and land-use classification showed some evidence of dry conditions after the 2015−2015 ENSO. We conclude with recommendations for management of non-native species to support regeneration of the native ecosystems of the Southern Line Islands, with implications for other Pacific islands.

## Introduction

Remote reef islands and atolls host unique species and ecosystems, and are highly vulnerable to climate change impacts. The Intergovernmental Panel on Climate Change (IPCC) identifies sea level rise, cyclones, rising oceanic and air temperatures, and changing patterns of precipitation as the key risk factors facing reef islands and atolls [1]. Although they will impact islands differently, there is high confidence in the expected detrimental impacts of these factors on islands globally [1]. Biodiversity on reef islands is threatened in numerous ways. Coral reef ecosystems are threatened by thermal stress [2,3]; coastal terrestrial ecosystems by sea level rise [4,5]; and inland terrestrial ecosystems from rising temperatures and drought conditions resulting in species range shifts [1,4]. As a result, the species and ecosystems on these islands are important to document and conserve in the face of the increasing severity of climate change impacts.

The biodiversity of low-lying, tropical reef islands and atolls is extremely vulnerable given projected sea level rise and climatic changes [4]. Reef islands and atolls may experience coastal erosion or inundation by future rising sea levels, and some of the unique coastal ecosystems may be lost or significantly modified [1]. These ecosystems are inherently dynamic with high turnover. For example, naturally variable sediment processes can result in changes in island geomorphic structure particularly around the shoreline [5,6] and storms can lead to shifts in ecological community structure and function [7–9]. Small reef islands and atolls are expected to experience shifts with climate change, in some cases expanding in area, and in some cases contracting due to changes in local eco-morphodynamics including wave-reef interactions and reef ecological processes [10,11]. Inland plant communities will be impacted by these geomorphic changes, as well as by changing temperatures and precipitation, which may be favourable, neutral, or detrimental depending on the species and island or atoll in question [12]. Research on plant species distributions on reef islands and atolls, specifically studies examining the impacts of recent and extreme weather patterns, are vital to help illuminate possible futures for these ecosystems.

A critical region to consider with respect to climate change impacts on reef islands and atolls is the Pacific Ocean, given that it is underrepresented in the literature, despite the sheer quantity of islands it encompasses [4,13,14]. The Pacific Ocean spans over 30% of the surface of the Earth [12,15]. The region hosts unique terrestrial biodiversity, with high rates of endemism and species diversity, and multiple biodiversity hotspots [4,14,15]. Sea level rise and increased severity of storms is anticipated to strongly impact terrestrial biodiversity and human populations in coastal regions throughout the Pacific [4,16]. Storms may be destructive for some Pacific Island ecosystems, and constructive for others such as through reworking and injection of sediment [8]. *Pisonia* trees, which are an important species in Pacific Island forests, have in some cases been shown to experience positive successional effects following storms [7,17]. Investigating these nuanced impacts of climatic events on the plant communities of Pacific Islands is valuable for understanding the implications for biodiversity conservation.

Among the least studied floras of the Pacific are the Southern Line Islands, a group of small uninhabited islands in the South Pacific. The group of islands includes Millennium Atoll (formerly known as Caroline Atoll and Caroringa), Flint Island, and Vostok Island. Each island has a unique geography, and climatological and oceanographic factors that impact the ecosystems of the islands [18]. Some archaeological evidence of small human settlements exists, namely on Millennium Atoll [19,20], and the islands have different histories of land-use and resource extraction [21]. The islands were primarily used for the extraction of guano, and for the establishment of coconut plantations for copra production in the 18^th^ and early 19^th^ centuries [19,22]. Today, the islands are protected under the jurisdiction of the government of the Republic of Kiribati and are included in the Southern Line Islands Marine Protected Area (SLIMPA), which also includes Starbuck and Malden islands. Owing to their remote location, the ecosystems of the Southern Line Islands have been released from human pressure for decades. While some studies have been conducted on the marine ecosystems in the Southern Line Islands, such as on coral reef ecosystems and marine microbial ecology [23–26], the current conditions of the terrestrial ecosystems of the islands are largely unknown.

Botanical research on the Southern Line Islands is limited to a few studies in the 19^th^ and 20^th^ centuries, which described the plant species on the islands, including species lists and qualitative notes on the botanical communities (Table 1). Terrestrial ecological or botanical studies have not been conducted in the 21^st^ century. Since these studies, two key processes have likely affected the extant plant communities: the abandonment of commercial uses, and the strong El Niño-Southern Oscillation (ENSO) of 2015–2016.

**Table 1:** Historic studies including vegetation surveys of Flint Island, Millennium Atoll, and Vostok Island. (NA: unpublished reports).

Location	Authors	Year of survey	Year published
Flint Island	H. St. John & F. R. Fosberg [22]	1934	1937
	A. K. Kepler and others with the International Council for Bird Preservation (ICBP) [27]	1990	NA
Millennium Atoll	F. D. Bennett	1835	1840
	W. S. Dixon, 1884 (made observations and plant collections) [28] Trelease, 1884 (published study) [29]	1884	1884
	Clapp & Sibley [19]	1965	1971a
	A. K. Kepler & C. B. Kepler, and others with the International Council for Bird Preservation (ICBP) [30]	1988, 1990	1994
Vostok Island	W. J. Anderson (made observations), F. R. Fosberg (published study) [31]	1935	1937
	Clapp & Sibley [32]	1965	1971b
	A. K. Kepler and others with the International Council for Bird Preservation (ICBP)	1990	NA

Phenomena related to El Niño-Southern Oscillation (ENSO) are known to impact many Pacific islands, resulting in fluctuations in rainfall and sea-surface temperature, which vary across islands [33]. In the Pacific, cold tongue El Niño and mixed El Niño tend to result in wetter than average conditions, while La Niña events tend to result in dry conditions in the Line Islands (including the Northern Line Islands and Southern Line Islands) [33,34]. Although there are no studies to date on the 2015–2016 ENSO effects in the Southern Line Islands, research elsewhere has shown that ENSO events can have strong effects on vegetation [35,36]. In addition, vegetation on small oceanic islands is sensitive to the impacts of ENSO given their dependence on marine systems [37]. Abundant research shows that seabirds provide critical nutrient inputs, where guano enhances soil health and plant productivity, yet their populations are impacted by climatic changes [38–41]. Thus, we anticipated that the presence of native seabirds in the Southern Line Islands, and the possible impacts of the ENSO event on seabird populations, would in turn impact the plant communities of the islands. Additionally, drought conditions in general have been shown to result in loss of biomass, productivity, and death of vegetation in Pacific Islands [42–44]. Further investigation, including utilizing climatic and remote-sensing data, is needed to understand the impacts of ENSO on the Southern Line Islands. Assessing satellite imagery in combination with on-the-ground vegetation surveys provides a more in-depth assessment of the impacts of climatic changes on terrestrial ecosystems of reef islands and atolls islands than using either method alone.

Finally,, it is important to examine natural regeneration and ecological succession on reef islands and atolls following release from commercial human activities, such as coconut plantations and guano extraction, to inform whether any active ecological restoration should be undertaken to support the native ecosystems post-disturbance. Ecological succession on reef islands and atolls has been described as circular or cyclical, due to historical patterns of emergence then submergence and continuously changing sediment deposits, which interact with biotic processes such as nutrient inputs by seabirds and colonization by land-crabs, and contribute to recurring patterns of plant species assembly [45]. When the natural processes of succession on reef islands and atolls are disturbed by the removal and introduction of plant species, the outcomes can be detrimental to these dynamic ecosystems. Studies have shown that coconut plantations, such as on Palmyra Atoll, result in reductions in native tree diversity, water uptake for native plants, and loss of soil macronutrients [46,47]. Other research on islands has shown that following the abandonment of plantation forestry or agriculture, many non-native, invasive species become dominant [48,49]. Further research in reef island and atoll terrestrial ecosystems would be valuable to provide information to support rehabilitation of native forest ecosystems following abandonment of coconut plantations [50]. Additionally, despite the economic boom of guano extraction and literature available on the history of guano extraction [51,52], or biological or chemical studies of guano [53–55], to our knowledge research is not available on ecological succession of plant communities post-guano extraction.

Research on the flora of the Southern Line Islands can help us to understand how plant communities on reef islands and atolls respond to climatic events and rebound after such histories of commercial land-use. National Geographic Pristine Seas Program conducted two expeditions to the Southern Line Islands in 2009 and 2021, which collected data on the vegetation and terrestrial ecosystems. In this paper, we use these data to characterize the vegetation to address the following questions for Millennium Atoll, Flint Island, and Vostok Island:

(i) How has island vegetation (species composition, richness, and cover) changed since the historic botanical surveys?
(ii) How does vegetation (plant frequency of occurrence, species composition, and cover) differ across the islands which have different geographies and land-use histories?
(iii) Are there differences in vegetation (plant frequency, composition, and cover) before (2009) versus after ENSO (2021), and does this vary across islands?

We hypothesized that succession has resulted in greater species richness and cover by non-native species on the islands with historic industrial land-use (Flint Island and Millennium Atoll). We expected there would be lower species richness on the smaller islands and islets, following the theory of island biogeography. Lastly, we expected a decrease in vegetation cover due to the ENSO-related dry conditions.

## Methods

### Study area

The Southern Line Islands—part of the Line Islands chain, composed of limestone seamounts, emerged limestone islands, and atolls [56]—range in size from Vostok (10°06’S, 152°23’W) at 0.25 km^2^, to Flint (11°25.43’S, 151°48’W) at 3.24 km^2^, to Millennium Atoll (10°00’S, 150°13.5’W) with a total area of 4 km^2^. Vostok, as the smallest, is triangular with a perimeter of sand beaches and coral rubble, and is located 3–4.6 m above sea level [30]. Flint is a coral island, with elevation up to approximately 8 m, and it also has a perimeter of sand beaches and coral rubble, with few small lagoons in the interior, formed following historic mining activities which have now filled with brackish water [57]. Millennium Atoll is a low coral ring comprised of three large islets (Nake and Long, which are the northern-most islets, and South, which is the southern-most islet), as well as approximately 36 smaller islets, with a large lagoon in the centre; the maximum elevation is 3 m [20,26]. The Southern Line Islands are mesic ecosystems, with average annual rainfall between 2008 and 2022 of 2050 mm for Flint, 2120 mm for Vostok, and 2130 mm for Millennium [58].

### Field methods

Vegetation surveys were conducted on Flint Island, Vostok Island, and all islets within Millennium Atoll. Our field research was conducted in the Southern Line Islands Marine Protected Area under Permit no. SRP1/21, Ministry of Environment, Lands and Agricultural Development of Kiribati. Field surveys were conducted at two time points for approximately one month each time, from 30 March to 3 May 2009, and October 2021. One of us (M.F.) conducted transects on the islands and islets. Transects were positioned to be semi-systematic in a zig-zag manner, while not on a grid, to provide representative coverage of the islands and islets. An effort was made to cover every vegetation and edaphic zone noted on satellite imagery, including small areas of interest such as ponds and clearings. In 2021, the transects were laid out to replicate the transects from 2009. Along the transects, relevé plots were conducted to record plant species occurrences and visually estimate species percent covers, using 10x10 m plots for woody plants and 1x1 m plots for herbaceous plants. Plots were not conducted at a set interval, but were positioned to capture vegetation types along the transect line. Other plant species outside the plots were also documented to determine total plant species richness on each island. Qualitative observations on the vigor of plant species were made. GPS locations of all individuals were taken, as were GPS tracks of the transects walked.

The sampling scheme of transects differed on each island/islet due to their different sizes and terrains. On Vostok, the three major vegetation zones (beach scrub, forest and forest clearings) were surveyed, with effort made to cover all major forest blocks of various age classes and states. On Flint, which is dominated by coconut palms, systematic transects were laid out to cover the high/dry edge and lower/wet internal areas by traversing the island laterally throughout, with a focus on capturing the zones which were regenerating with native vegetation and ponds. On Millennium, an effort was made to cover every islet on the survey going from ocean side to the lagoon side. On Vostok Island, four transect lines were walked; on Millennium Atoll, one to two transect lines were walked on each islet (depending on their size); on Flint Island, 11 transect lines were walked. In 2021, the transects from 2009 were repeated using the past GPS tracks.

### Analysis of vegetation characteristics

To compare plant species richness over time, we documented and compared the species found in the historic studies to those found in 2009 and 2021. For the 2009 and 2021 surveys, we included all species found in all areas of the islands. We generated species accumulation curves for Flint Island and Millennium Atoll (but not for Vostok Island as the number of plots and species were low) in the package vegan in R Studio (v. 4.4.0), to ensure that the curves plateaued and we had successfully detected all species (S1 Fig).

We calculated species frequency (the number of plots in which species were found) across islands. We then took a subset of the plots that occurred on the same transects or in the same area (within 50 m) in both 2009 and 2021 for each of Flint Island, Vostok Island, and Millennium Atoll, and limited the frequency calculations to the subset of the plots. In this way, we ensured the sampling effort was equivalent between the two years, and we were able to compare species frequencies across the islands over time. The number of plots for Flint Island was 69 from 2009 and 85 from 2021; for Millennium Atoll, 167 plots from 2009 and 179 from 2021; for Vostok Island, 10 plots from 2009 and 8 from 2021.

To test if there were changes in species cover and composition between 2009 and 2021, and to determine whether native and non-native species had changed in cover over time within an island, we also used the subset of the plots per island. We calculated the mean percent cover for each species across the subset of the plots, for each island and year. To assess differences in species cover across years for each island, due to heteroscedasticity and non-normality of the data, we used Wilcoxon rank-sum tests for unpaired data (as we monitored in the same areas, but not the same plots) with cover against year, for each species on each island.

To compare species composition across islands and years, we used Non-Metric Multidimensional Scaling (NMDS) ordination. We summed the number of individuals of each species across the subsample of plots in each transect. We ran the ordinations using the function metaMDS in the package vegan in R, with horn distance as this dissimilarity index accommodates differences in sampling effort across plots. To assess the differences in composition across islands, we conducted Multi Response Permutation Procedure (MRPP) models, using the function mrpp with Bray distance and 999 permutations, also in the package vegan. Lastly, we used the package betapart to assess beta-diversity (function beta.sor for the Sørensen dissimilarity index), species turnover (function beta.sim for the Simpson dissimilarity index), and species nestedness (function beta.sne), where beta.sor is the sum of beta.sim and beta.sne.

### Analysis of remote sensing imagery and climatic changes

To assess the impacts of ENSO on vegetation between 2009 and 2021, and to compare across islands, we determined changes in the normalized difference vegetation index (NDVI) and conducted supervised classifications of satellite imagery for Flint Island, Vostok Island, and Millennium Atoll. Remote sensing data are commonly used for vegetation mapping and monitoring, and NDVI is one of the most common indices used to study vegetation dynamics [59,60]. This index is calculated using the Red and Near Infrared bands (NIR) and can be used to determine plant health, presence and absence of green vegetation and as a proxy for a number of other factors [59,61,62].

We selected high resolution remote sensing images from WorldView-2, WorldView-3, IKONOS, and GeoEye-1. Due to images being unavailable during the same months when the field surveys were conducted in 2009 and 2021, we used images taken from as close to the time periods of the field surveys as possible, and with minimal cloud cover (less than or equal to 10%) to make analyses feasible. Since differences across years could be confounded by differences in seasons (e.g., spring and fall with the months of March versus September), for Millennium Atoll, we compared NDVI in two different seasons within the same year; we analyzed an image from March 2009 (one month prior to field sampling), and for 2021 we compared imagery from March and December (two months after sampling). For Flint Island, we analyzed an image from March 2009 (same month as sampling) and September 2021 (one month prior to sampling). For Vostok Island, we analyzed an image from September 2010 (17 months after sampling) and from March 2022 (five months after sampling), as these were the closest available images to the 2009 and 2021 sampling periods respectively.

NDVI was calculated using the standard formula for all three islands. NDVI calculation was performed per cloud masked image using the standard methodology, using Google Earth Engine (GEE) with the JavaScript API [63,64]. NDVI was then added as an additional band to the images to maintain consistency within the project. Images were converted to raster data and stacked in R. These stacks of values were then converted to tabular format where each pixel was defined and assigned its NDVI values. All pixels that were masked via the cloud masking algorithm developed specifically for this dataset were omitted.

NDVI values below 0.4 were not included in the analysis due to our emphasis on terrestrial vegetation cover. NDVI values between 0.2–0.5 generally indicate the detection of vegetation or low vegetation cover, while some studies indicate that medium to dense vegetation cover begins around 0.4 [60,65,66]. Furthermore, our dataset covers a unique set of islands and atolls, some of which have variable coastline that can expose wet rocks, algae and other marine features, which can have significant increases in NDVI values depending on the time at which the satellite image was captured [67]. The coastline variability resulted in values in our analysis in areas that were visually covered with water—which in theory should have either a negative value or values close to 0—to fluctuate up to 0.2 or higher in some cases. This may have been due to reflectance, which could be indicative of exposed algae [68]. This noise was reflected in the distribution of plots and was not indicative of what was visually present in the forested regions, nor did it change the averages of the NDVI significantly. Therefore, the values below 0.4 were not included in the analysis, to provide a smoother and more accurate representation of forested vegetation conditions on the islands. Tabular data were merged from all islands and used to develop density plots of NDVI values for each island in R. Density plots for each island were developed and analyzed for changes in NDVI values pre- (2009 for Flint and Millennium, 2010 for Vostok) and post-ENSO (2021 for Flint and Millennium, 2022 for Vostok).

With the remote sensing images, we then conducted land-cover classifications for each island, for each image, based on the NDVI values. Images were screened and cloud masks were applied to remove high elevation cloud contamination in pixels within the Flint 2009, Millennium March 2009, and Millennium December 2021 images in order to provide more consistent band values and remove potential outlying values. Due to the differences in the images (e.g., different satellites, different cloud cover, etc.), within image classification techniques were utilized. NDVI values remained consistent between images, and all images were resampled to two meters for the categorical classification of land-cover. Therefore, the land-cover categories developed could be used across all islands in the study consistently. Land-cover classification was determined using NDVI and the red, green, blue (RGB), and near-infrared (NIR) bands in the image. NDVI thresholds drove the majority of the land-cover analysis and were developed after visually examining all images for broadly recognized categories. These categories were present in other classification schema or were visually recognized as being unique to the images. Broad categories used for initial analysis were defined as intertidal or water-covered shoreline, sand, scrub, and closed-canopy. These categories were captured by randomly placed polygons that contained the category in question throughout each image at the discretion of the technician. Polygons were then divided into individual pixels and band values were extracted.

The final list of discrete zones was informed by several commonly used thresholds in remote sensing practice as well as observations of the imagery (S1 Table). Water values generally fall between −1 and 0, therefore areas that experienced some water coverage due to tidal inundation and seasonal variability needed to be captured to train the classification. Sand values were defined to better capture fluctuating shorelines over the course of the study, and to exclude it from areas where vegetation may be present. Sand categories were defined visually as sand can change color and reflectance based on if it is wet or dry, which can skew its values. The scrub category initially was based on common practice in remote sensing that defines senescing or dry vegetation as values between 0.2–0.5. These base values were further divided, based on observations within the image and previous experience with island ecological zones, into “beach scrub layer 1” and “beach scrub layer 2.” Beach scrub layer 1 captures transitional shoreline zones with sparse vegetation and low NDVI. Generally, shoreline zones in the South Pacific are dominated by beach scrub, grasses, and small hardy plants. These plants, which are generally small or sparse have low NDVI due to direct exposure to sunlight and harsh, salt-heavy conditions. As distance from the shoreline increases, these plants generally transition to denser, more established stands and will generate overall higher NDVI values, which we classified as “beach scrub layer 2.” Upon visual inspection, the separation of these categories additionally provided more context to the land-cover map and more accurately depicted the high variability and mosaiced land-cover over the islands and the atoll. The open-canopy category was developed to capture vegetation with NDVI values of 0.5–0.7. This term may be misleading as these values can be found within closed-canopy regions and are highly dependent on seasonality. However, given the general trend in this high-resolution imagery of capturing the spatial nuance on these atolls and the changing density of foliage, this category adequately capturing forested areas near the exterior of the atolls or on small islets such as is the case on Millennium. The closed-canopy category from 0.7–1 was specifically developed to understand where vegetation had the highest NDVI within the interior of the islands. In this particular case, these high NDVI zones also correspond to areas of high plant density or what was deemed closed-canopy. However, high NDVI does not always correlate to closed-canopy and this term may not be suitable for other studies.

The zones were defined for each image, then visually inspected for adherence to the defined values using NDVI colored rasters. The zones were then vectorized to create polygons containing all pixels within a region that contained the category at a two-meter resolution. Vectors were then separated based on category value to be used in training data. Training data was developed using as many vectors and points as was computationally possible. In this way, 300–600 random vectors within a category were chosen and from those random vectors, 500–1000 points were chosen as the input into the training data. This randomization was completed for each of the five categories developed. After all points were created, they were merged into a list and split into 70% training data and 30% testing data. This data was inputted into a 500-tree Random Forest model and trained on the NDVI band as well as the RGB and NIR bands within the images. Accuracy metrics such as importance values, out of bag error estimates, error matrices, consumers accuracy, producers’ accuracy, and kappa test validation were run on both the training and testing data sets for each image. On average, producers and consumers accuracy ranged from 85–99% demonstrating the effectiveness of the classification (S2 Table).

Lastly, we gathered and summarized data on mean monthly precipitation for the islands (S2 Fig). Data was sourced from the Climate Hazards Center of the University of California Santa Barbara CHIRPS 2.0 global-2 monthly data on precipitation [58]. We included the years 2008–2022 for each island, to capture the months and years when the field surveys were conducted, when the remote sensing images were taken, the year prior to the start of the first field survey (2008), and the year following the last field survey (2022, which was also the year of the Vostok satellite image). We used bounding boxes with the maximum and minimum longitudes for Flint Island, Vostok Island, and Millennium Atoll, and extracted the daily values for each year, then calculated the mean monthly precipitation values. For Flint and Vostok respectively, one pixel of CHIRPS data was used, and for Millennium, two pixels were used. Data from the database was not available for January or December 2017 for any location. We compared rainfall values between March and September 2021, corresponding with the investigation into possible differences in NDVI values for Millennium Atoll, to validate our comparison between the remote sensing imagery taken from different seasons.

## Results

### Changes in vegetation characteristics since historic studies

Since the historic botanical studies in the 20^th^ century and earlier, species richness and composition showed changes on some of the islands more than others (Table 2). After the release of human pressure with the abandonment of the coconut plantations and termination of guano harvesting on the islands, ecological succession proceeded over time. The largest difference observed in the 2009 and 2021 surveys was a reduction in introduced species compared to the historic studies. This was most notable on Millennium Atoll and Flint Island.

**Table 2:** Vascular plant species documented on Millennium Atoll, Flint Island, and Vostok Island in previous studies (dates indicate date of publication) and the current study from 2009 and 2021. Presence detected in a study denoted with 1, and 0 indicates the species was not found. We denote species found in our study that were not previously found with the color blue, and species absent in our study that were previously found with the color red, pertaining to each island/atoll separately.

Species	Growth form	Millennium Bennett 1840	Millennium Dixon, reported by Trelease 1884	Millennium Clapp & Sibley 1971	Millennium Kepler & Kepler 1994	Millennium 2009	Millennium 2021	Flint Fosberg & St. John 1937	Flint Kepler 1993	Flint 2009	Flint 2021	Vostok Fosberg 1937	Vostok Clapp & Sibley 1971	Vostok Kepler 1990	Vostok 2009	Vostok 2021
**Native Species**																
*Boerhavia repens* L.	Herb	1	1	1	1	1	1	0	1	1	1	1	1	1	1	1
*Boerhavia tetrandra* G.Forst. (listed as synonym *Boerhavia diffusa* L. variety *tetrandra* (Forst.) Heim. by Fosberg & St. John)	Herb	0	0	0	0	0	0	1	0	0	0	0	0	1	0	0
*Colubrina asiatica* Brongn.	Shrub	0	0	0	0	1	1	0	0	0	0	0	0	0	0	0
*Cordia subcordata* Lam.	Tree	0	1	1	1	1	1	1	1	1	1	0	0	0	0	0
*Cyperus javanicus* Houtt. (listed as synonym *Cyperus pennatus* Lam. by Fosberg & St. John)	Herb	0	0	0	0	0	0	1	1	0	0	0	0	0	0	0
*Digitaria* sp.	Grass	0	1	1	1	0	0	0	0	0	0	0	0	0	0	0
*Euphorbia* sp.	Herb	0	0	0	0	0	0	0	0	1	1	0	0	0	1	1
*Guettarda speciosa* L.	Shrub	0	0	0	0	1	1	1	1	1	0	0	0	0	0	0
*Heliotropium anomalum* Hook. & Arn.	Shrub	1	1	1	1	1	1	0	0	0	0	0	0	0	0	0
*Heliotropium arboreum* (Blanco) Mabb. (listed as synonyms *Tournefortia argentea* L.f. or *Messerschmidia argentea* (L.) in previous studies)	Shrub	1	1	1	1	1	1	1	1	1	1	0	0	0	1	1
*Hernandia nymphaeifolia* (C.Presl) Kubitzki	Tree	0	0	0	0	1	1	0	0	0	0	0	0	0	0	0
*Hibiscus tiliaceus* L.	Tree	0	0	0	1	0	0	0	1	1	1	0	0	0	0	0
Inocarpus fagifer (Parkinson ex F.A.Zorn) Fosberg (listed as synonym *Inocarpus fagiferus* (Park.) Fosberg by Bennett)	Tree	1	0	0	0	0	0	0	0	0	0	0	0	0	0	0
*Laportea aestuans* (L.) Chew	Herb	0	0	0	0	1	1	0	0	1	1	0	0	0	0	0
*Laportea ruderalis* (G.Forst.) Chew (listed as synonym *Fleurya ruderalis* Gaudich in previous studies)	Herb	1	1	1	1	0	0	1	1	0	0	0	0	0	0	0
*Lepidium bidentatum* Montin	Herb	1	1	0	1	0	0	1	0	1	1	0	0	0	0	0
*Lepturus repens* (G.Forst.) R.Br.	Herb	0	1	1	1	1	1	1	0	1	1	0	0	0	0	0
*Microsorum scolopendria* (Burm.f.) Copel. (listed as synonyms *Polypodium scolopendria* Burm.f. or *Phymatosorus scolopendria* (Burm.f.) Pic.Serm. in previous studies)	Fern	1	1	1	1	1	1	1	1	1	1	0	0	0	0	0
*Nephrolepis* sp.	Fern	0	0	0	0	0	1	0	0	0	0	0	0	0	0	0
*Pandanus* sp.	Tree	0	0	0	0	1	1	0	0	1	1	0	0	0	0	0
*Pandanus* *tectorius* Parkinson	Tree	1	1	1	1	0	0	0	1	0	0	0	0	0	0	0
*Pandanus utilis* Bory (listed as synonym *Pandanus odoratissimus* Jacq. by Fosberg & St. John)	Tree	0	0	0	0	0	0	1	0	0	0	0	0	0	0	0
*Phyllanthus amarus* Schumach. & Thonn.	Herb	0	1	1	1	0	0	0	0	0	0	0	0	0	0	0
*Pisonia grandis* R.Br.	Tree	0	1	1	1	1	1	1	1	1	1	1	1	1	1	1
*Portulaca oleracea* L. (listed as synonym *Portulaca fosbergii* Von Poelln. by Fosberg & St. John)	Herb	0	0	0	0	0	0	1	1	0	0	0	0	0	0	0
*Portulaca lutea* Sol. ex G.Forst.	Herb	1	1	1	1	1	1	0	0	0	0	0	0	0	0	0
*Psilotum nudum* (L.) P.Beauv.	Whisk fern	0	0	1	1	0	0	1	1	0	0	0	0	0	0	0
*Scaevola taccada* (Gaertn.) Roxb.	Shrub	0	0	0	1	0	0	0	0	0	0	0	0	0	0	0
*Sida fallax* Walp.	Shrub	0	1	0	1	0	0	0	0	0	0	0	0	0	0	0
*Suriana maritima* L.	Shrub	0	1	1	1	1	1	0	0	0	0	0	0	0	0	0
*Terminalia catappa* L.	Tree	0	0	0	0	0	0	0	1	1	1	0	0	0	0	0
*Thespesia populnea* Sol. ex Corrêa	Tree	0	0	0	1	0	0	1	1	1	1	0	0	0	0	0
*Tribulus cistoides* L.	Herb	0	0	1	1	1	0	0	0	0	0	0	0	0	0	0
**Historic Polynesian Introductions**																
*Morinda citrifolia* L.	Tree	1	1	1	1	1	1	1	1	1	1	0	0	0	0	0
*Tacca leontopetaloides* (L.) Kuntze	Herb	1	0	1	1	0	0	0	1	0	0	0	0	0	0	0
**Recent Introductions**																
*Achyranthes aspera* L.	Herb	0	0	0	0	1	1	0	0	0	0	0	0	0	0	0
*Ananas comosus var. microstachys* (Mez) L.B.Sm. (listed as *Ananas comosa* (L.) by Dixon)	Herb	0	1	0	0	0	0	0	0	0	0	0	0	0	0	0
*Anredera cordifolia* (Ten.) Steenis (listed as synonym *Boussingaultia gracilis* Miers forma *pseudo-basseloides* Hauman by Dixon, Trelease)	Herb	0	1	0	0	0	0	0	0	0	0	0	0	0	0	0
*Artocarpus altilis* (Parkinson) Fosberg (listed as *Artocarpus incisa,* synonym of *Artocarpus incisus* (Thunb. L.f.) by Fosberg & St. John)	Tree	0	0	0	0	0	0	1	1	0	0	0	0	0	0	0
*Bidens pilosa* L.	Herb	0	0	0	0	0	0	1	0	0	0	0	0	0	0	0
*Brassica rapa subsp. oleifera* (DC.) Metzg. (listed as synonym *Brassica campestris* L. by Fosberg & St. John)	Herb	0	0	0	0	0	0	1	0	0	0	0	0	0	0	0
*Calophyllum inophyllum* L.	Tree	0	1	0	0	1	1	1	1	1	1	0	0	0	0	0
*Cannabis sativa* L.	Herb	0	0	0	0	1	0	0	0	0	0	0	0	0	0	0
*Capsicum frutescens* L.	Shrub	0	0	0	0	0	0	1	0	0	0	0	0	0	0	0
*Carica papaya* L.	Tree	0	1	0	0	1	0	1	1	0	0	0	0	0	0	0
*Citrus* x *aurantium* nothof. aurantium	Tree	0	0	0	0	0	0	1	0	0	0	0	0	0	0	0
*Cocos nucifera* L.	Palm	1	1	1	1	1	1	1	1	1	1	0	0	0	0	0
*Crinum asiaticum* L.	Herb	0	0	0	0	0	0	1	1	1	1	0	0	0	0	0
*Crinum* sp.	Herb	0	1	0	0	0	0	0	0	0	0	0	0	0	0	0
*Cucurbita pepo* L.	Herb	0	1	0	0	0	0	0	0	0	0	0	0	0	0	0
*Cyanthillium cinereum* (L.) H.Rob. (listed as synonym *Vernonia cinerea* (L.) Less. by Fosberg & St. John, and Kepler)	Herb	0	0	0	0	0	0	1	1	0	0	0	0	0	0	0
*Cyperus mindorensis* (Steud.) Huygh (listed as synonym *Kyllinga cephalotes* (Jacq.) Druce by Fosberg & St. John)	Sedge	0	0	0	0	0	0	1	0	0	0	0	0	0	0	0
*Eleusine indica* (L.) Gaertn.	Grass	0	1	0	0	0	0	0	0	0	0	0	0	0	0	0
*Eragrostis tenella* (L.) P.Beauv. ex Roem. & Schult.	Grass	0	1	0	0	0	0	0	0	0	0	0	0	0	0	0
*Eragrostis viscosa* (Retz.) Trin. *(listed as synonym Eragrostis amabilis* (L.) Wight & Arn. by Fosberg & St. John)	Grass	0	0	0	0	0	0	1	0	0	0	0	0	0	0	0
*Euphorbia hypericifolia* L.	Herb	0	0	0	0	0	0	1	0	0	0	0	0	0	0	0
*Euphorbia parviflora* L. (listed as synonym *Euphorbia pilulifera* L. by Dixon, Trelease)	Herb	0	1	0	0	0	0	0	0	0	0	0	0	0	0	0
*Ficus carica* L.	Tree	0	1	0	0	0	0	1	1	0	0	0	0	0	0	0
*Gardenia* sp.	Shrub	0	0	0	0	1	0	0	0	1	1	0	0	0	0	0
*Hibiscus rosa-sinensis* L.	Shrub	0	0	0	0	0	0	1	1	0	0	0	0	0	0	0
*Ipomoea batatas* (L.) Lam.	Herb	1	0	0	0	0	0	0	0	0	0	0	0	0	0	0
*Ipomoea pes-caprae* (L.) R.Br. (listed as synonym *Ipomoea pes-caprae* ssp*. brasiliensis* (L.) Ooststr. by Clapp & Sibley)	Herb	0	0	1	0	0	0	0	0	0	0	0	0	0	0	0
*Ipomoea violacea* L. (listed as synonym *Ipomoea glaberrima* Bojer ex Hook by St. John & Fosberg, *Ipomoea macrantha* Roem. & Schult. by Kepler & Kepler, and *Ipomoea tuba* (Schlecht.) G. Don by Clapp & Sibley)	Herb	0	0	1	1	1	1	1	1	0	0	0	0	0	0	0
*Ipomoea* sp.	Herb	0	0	0	0	0	0	0	0	1	1	0	0	0	0	0
*Leucaena leucocephala* (Lam.) de Wit	Tree	0	0	0	0	0	0	0	1	0	0	0	0	0	0	0
*Malvastrum coromandelianum* (L.) Garcke	Shrub	0	0	0	0	0	0	1	0	0	0	0	0	0	0	0
*Musa* x *paradisiaca* L. (listed as *Musa sapientum* L. by Fosberg & St. John)	Tree-like herb	0	0	0	0	0	0	1	0	0	0	0	0	0	0	0
*Paspalum dissectum* (L.) L. (listed as synonym *Paspalum vaginatum* Elliot by Kepler)	Grass	0	0	0	0	0	0	0	1	0	0	0	0	0	0	0
*Phyllanthus niruri* L.	Herb	0	0	0	0	0	0	1	0	0	0	0	0	0	0	0
*Russelia equisetiformis* Schltdl. & Cham.	Subshrub	0	1	0	0	0	0	0	0	0	0	0	0	0	0	0
*Solanum lycopersicum L.* (listed as synonym *Lycopersicum esculentum* Mill. by Fosberg & St. John)	Herb	0	0	0	0	1	0	1	0	0	0	0	0	0	0	0
*Synedrella nodiflora* Gaertn.	Herb	0	0	0	0	0	0	1	0	0	0	0	0	0	0	0

Millennium Atoll is the most species-rich of the Southern Line Islands; it has the greatest total area and greatest diversity of habitats across the islets. There have been few botanical studies on the atoll in the past. First, there was a study by Bennett (1840), in which 13 species were found in total, 9 of which were native; there was also a study by Dixon [28], reported by Trelease (1884) [29], in which 28 species were found, 15 of which were native. These historical studies were described by Clapp and Sibley (1971a); the original documents were not available. Clapp and Sibley (surveyed 1965, published 1971) documented 20 species, 17 of which were native. Lastly, Kepler and Kepler (surveyed in 1988 and 1990, published 1994) identified 26 species, 23 of which were native. Most of the native species we found in 2009 and 2021 were found previously, while some of the previously found cultivated or introduced species were not found in the current study. In our study, 24 species were found in 2009, and 20 species were found in 2021 (15 of which were native in both years).

The only historic botanical study we found for Flint Island was conducted by Harold St. John and F. Raymond Fosberg (surveyed in 1934, published in 1937), in which 36 vascular plants were found, 15 of which were native. The ICBP expedition in 1990 located 27 plant species [27,69]. In our 2009 survey, 20 vascular plant species were found, 14 of which were native; in 2021, 19 species were found, 13 of which were native. We found fewer introduced species on Flint Island than noted in the historic studies.

Few vascular species occur on Vostok. Only two species were noted in previous studies by Clapp & Sibley (1971b) [32], and in their study, they summarized the prior study published by Fosberg (1937) [31], which was a research note based on the observations and plant collections conducted by Captain W. J. Anderson in 1935. The Clapp & Sibley (1971b)report points out that Fosberg (1937) erroneously identified *Boerhavia repens* as *B. diffusa.* In the 1990 expedition, only *Boerhavia repens, Boerhavia tetrandra,* and *Pisonia grandis* were found (Kepler, 1990). In our study, four species were found, all of which were native; *Heliotropium arboreum* and *Euphorbia* sp. were not previously found on Vostok.

### Differences in vegetation characteristics across the islands

Flint Island, Vostok Island, and Millennium Atoll are of different sizes, have different histories of land-use, and show different plant species richness based onthe historic studies and the vegetation surveys in the present study (Table 3).

**Table 3:** Summary of island histories and characteristics for Flint Island, Millennium Atoll and Vostok Island from historic studies and current study with surveys in 2009 and 2021. Values represent the total number of species listed from each survey, as well as the number of native species in parentheses.

			Total number of species found in respective studies (with number of native species in parentheses)							
Island	Land area	History of land-use	Bennett 1840	Dixon, Trelease 1884	Fosberg, 1937	Clapp & Sibley, 1971	Kepler 1990, 1993	Kepler & Kepler, 1994	Current study, 2009	Current study, 2021
Flint	3.25 km^2^	Coconut plantations and minor guano mining. Polynesian settlements unknown.	NA	NA	36 (15)	NA	27 (14)	NA	20 (14)	19 (13)
Millennium Atoll	4 km^2^	Guano extraction and coconut plantations for copra production. Ancient Polynesian settlements, with 50 documented sites. Uninhabited when Europeans first visited in 1606 (Kepler & Kepler, 1994).	13 (9)	28 (15)	NA	20 (15)	NA	24 (20)	24 (15)	20 (15)
Vostok	0.25 km^2^	None documented. Old anchor and parts of guano era shipwreck found. Evidence of damage to the reef. Stones placed in what resembled Polynesian Marae were found. Native *Pisonia* forest remains.	NA	NA	2 (2)	2 (2)	3 (3)	NA	4 (4)	4 (4)

### Plant frequency and cover differences between 2009 and 2021

**Fig 1:**
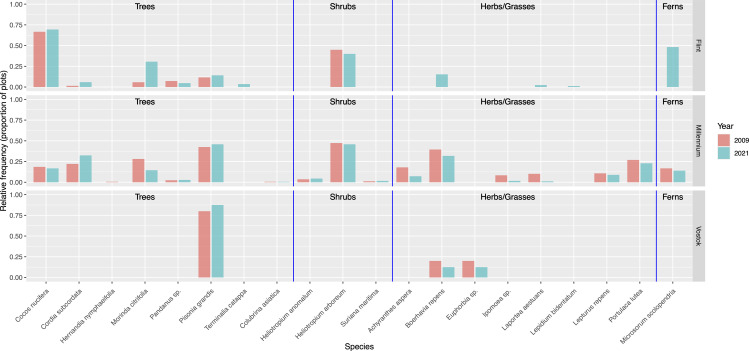
Relative frequency of species (proportion of plots in which species were found, in areas sampled both years) for 2009 and 2021, for Millennium Atoll and Flint and Vostok islands. Species organized into sections denoted by blue vertical lines for: trees, shrubs, herbs/grasses, ferns. Note that *H. arboreum* is not included in this Fig for Vostok because it was observed outside the survey plots.

Across the islands, species frequencies were similar across years with few differences (S3 Table, S4 Table, S5 Table). Some species present in 2009 were not found in 2021, or vice versa (Fig 1). For Flint Island, species not found in 2009 but found in 2021 in the same areas of sampling included *Boerhavia repens, Laportea aestuans, Lepidium bidentatum, Microsorum scolopendria* and *Terminalia catappa* (all of which are native species)*. Morinda citrifolia* (historic Polynesian introduction), *Cocos nucifera* (recent introduction) and *Pisonia grandis* (native species) had higher frequencies in 2021 than 2009, while all other species showed similar frequencies (within 10%) across the years. On Millennium Atoll, *Cordia subcordata* (native species) had a higher frequency in 2021 than 2009, while *Achyranthes aspera* (recent introduction), *Boerhavia repens*, *Ipomoea* sp. (recent introduction), *Laportea aestuans,* and *Morinda citrifolia* had higher frequencies in 2009 than 2021. Only one species, *Hernandia nymphaeaefolia* (native species) was not found in both years (only found in 2009). On Vostok, three species, *Pisonia grandis*, *Boerhavia repens*, and *Euphorbia* sp. (all native species) were found in the plots monitored both years, and the frequencies were similar (within 10%) across years. Note that a fourth species, *Heliotropium arboreum* (native species) was located outside of the relevé plots on Vostok (discussed below).

**Fig 2:**
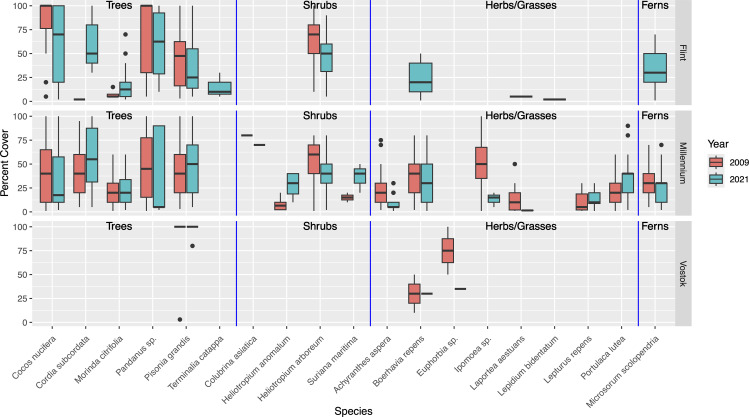
Percent cover of species on Flint Island, Millennium Atoll, Vostok Island in 2009 versus 2021. Species organized into sections denoted respectively by blue vertical lines for: trees, shrubs, herbs/grasses, ferns.

**Fig 3:**
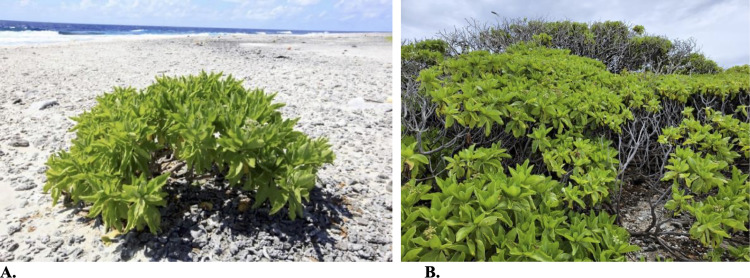
Single *Heliotropium arboreum* individual located outside of the relevé plots on the coastline of Vostok Island. The same individual is pictured in (A) from 2009, and (B) from 2021; photographs show the growth of the individual.

There were also relatively few differences across years in mean cover of species (Fig 2). For Flint Island, *Cocos nucifera* and *Heliotropium arboreum* cover decreased significantly between 2009 and 2021 (S6 Table). On Millennium Atoll, *Achyranthes aspera* and *Heliotropium arboreum* cover decreased significantly over time, while *Cordia subcordata, Heliotropium anamolum* (native species), and *Portulaca lutea* (native species) cover increased significantly (S7 Table). On Vostok Island, there were no differences in species cover over time from the three species noted in the plots (S8 Table). We did, however, document *Heliotropium arboreum* separately from the plots as it was only present in one location on the eastern shore of the island. In 2009, there was one individual of *Heliotropium arboreum* 60 cm in width, whereas in 2021, this individual had grown substantially into a large, many-stemmed individual (Fig 3).

### Plant species composition between 2009 and 2021

**Fig 4:**
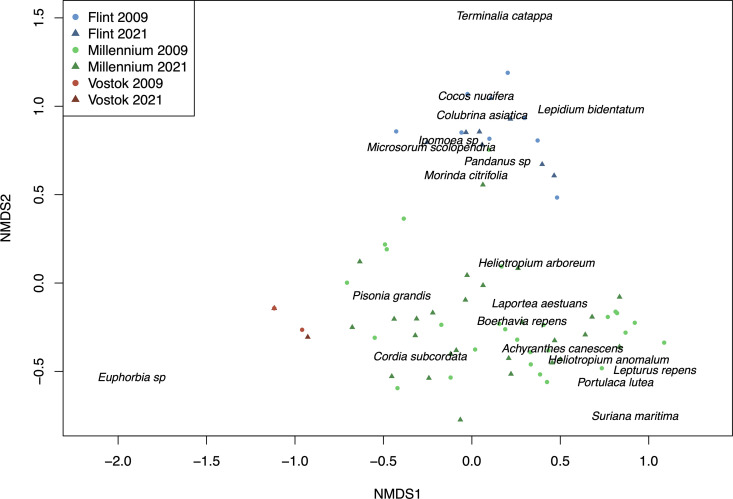
Non-metric multidimensional scaling (NMDS) ordination showing species compositions for each island for 2009 and 2021 with dominant species shown on the plot. Each point represents the species in all plots of one transect of an island. Ordination constructed in R using the function metaMDS in the package vegan, with 999 tries, horn distance, k = 3, and stress value = 0.097.

Species composition differed significantly between the islands (Fig 4), with p = 0.001 for each combination of Flint versus Vostok (effect size A = 0.389), Flint versus Millennium (A = 0.144), and Vostok versus Millennium (A = 0.127). *Colubrina asiatica, Heliotropium anomalum,* and *Suriana maritima* were shrubs only found on Millennium Atoll, and *Portulaca lutea* and *Tribulus cistoides* were herbs only found on Millennium. *Hibiscus tiliaceus, Lepidium bidentatum, Terminalia catappa* and *Thespesia populnea* were only found on Flint Island. One plot on Vostok in 2009 was an outlier (with only one individual of *Euphorbia* sp.) and was removed from the ordination plot.

In terms of beta-diversity, from 2009 to 2021 Flint had low dissimilarity, while Millennium and Vostok both had no dissimilarity between the years (S9 Table). Flint versus Millennium in 2009 and 2021 had moderate dissimilarity with 54% and 67% of species shared respectively. Millennium versus Vostok had lower dissimilarity, with 21% species shared for both years. In terms of species turnover from 2009 to 2021 within island, each of Flint, Millennium and Vostok had no species turnover. Flint versus Millennium in 2009 and 2021 had low species turnover; Flint versus Vostok in 2009 and 2021 had moderate to high turnover; Millennium to Vostok had moderate turnover both years. In terms of species nestedness, for Flint between 2009 and 2021, the nestedness value was low, while Millennium and Vostok both had all species the same in the two years (nestedness value of zero). For Flint versus Millennium, 2009 and 2021 had moderate to low nestedness respectively. For Flint versus Vostok, 2009 and 2021 had low to moderate nestedness respectively. For Vostok versus Millennium, the nestedness in both years was moderate. Finally, comparing all three islands, beta-diversity was moderate, turnover was low, and nestedness was low in both 2009 and 2021, with slightly lower values for each in 2021 than in 2009.

### Evidence of dry conditions between 2009 and 2021

**Fig 5:**
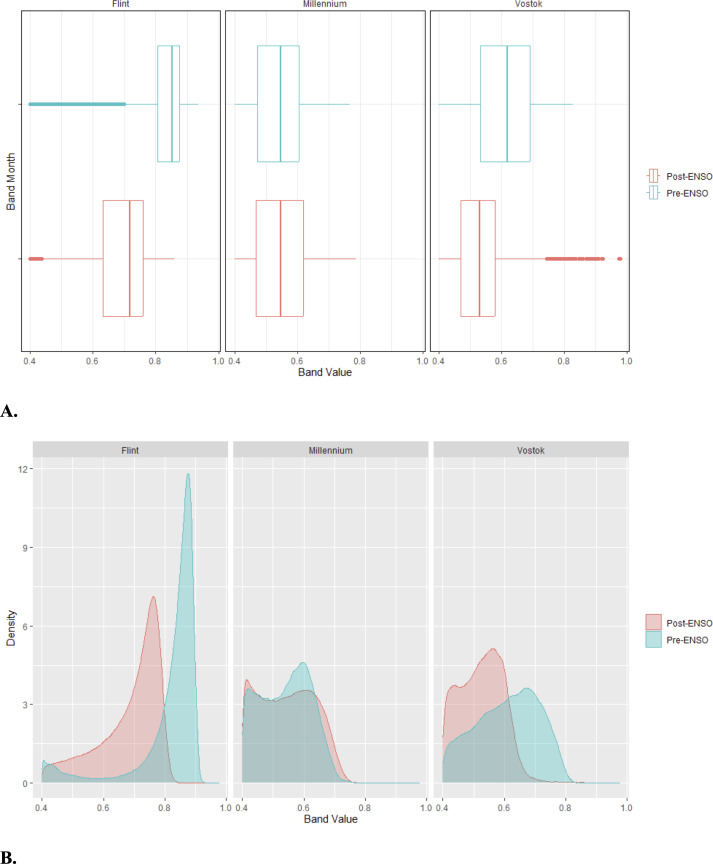
Boxplots (A) and density plots (B) showing the range of NDVI values for the pixels of the high-resolution remote sensing images for Flint Island, Millennium Atoll, and Vostok Island before and after the El Niño-Southern Oscillation (ENSO) event. The dates of the Pre-ENSO imagery used for the analysis included March 2009 for Flint and Millennium, and September 2010 for Vostok, while the post-ENSO imagery included September 2021 for Flint Island, December 2021 for Millennium Atoll, and March 2022 for Vostok.

NDVI values showed different trajectories across the islands when the pre-ENSO (2009, 2010 for Vostok) and post-ENSO (2021, 2022 for Vostok) remote sensing images were compared (Fig 5). On Flint Island, NDVI values decreased post-ENSO, indicating lower greenness. On Millennium Atoll, NDVI values showed no significant changes pre- versus post-ENSO. On Vostok, the lower quartile, median, and upper quartile post-ENSO were lower than the median pre-ENSO, yet several outlier pixels post-ENSO showed higher NVDI values.

**Fig 6:**
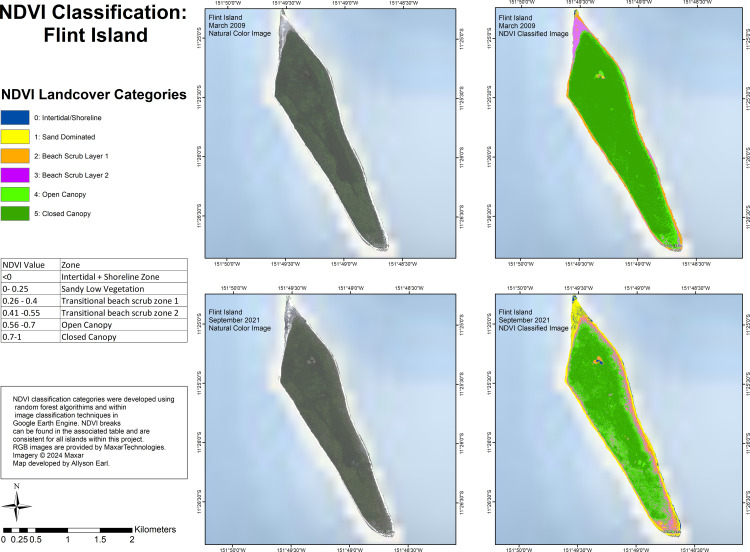
Land-cover classification using NDVI values for Flint Island for imagery from March 2009 and September 2021.

Vegetation classification based on NDVI showed differences within islands across the years. For Flint Island (Fig 6), open-canopy showed a greater extent in 2021 compared to 2009 when closed-canopy dominated the island. In 2021, the eastern coast and southern point of the island showed a thick cover by beach scrub layer 1, beach scrub layer 2, and sand. The northern point of the island, which in 2009 had been covered with beach scrub layer 2, was covered with sand with small speckles of intertidal + shoreline in 2021**.**

**Fig 7:**
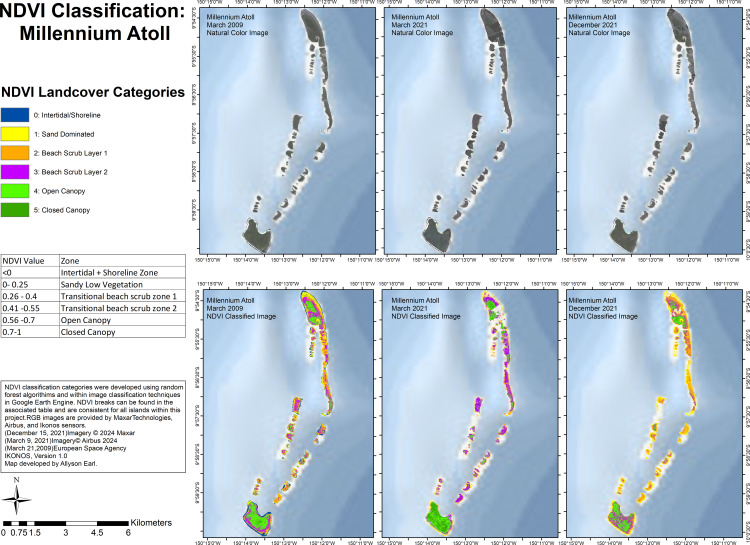
Land-cover classification using NDVI values for Millennium Atoll for imagery from March 2009, March 2021 and December 2021.

For Millennium Atoll (Fig 7), in 2009, Nake Islet, Long Islet, and South islet, as well as the majority of the other islets showed a combination of most of the land-cover types. Open-canopy dominated on Nake, Long and South islet in 2009, while closed-canopy was nearly absent. In 2021, changes across the year were seen: in March 2021, beach scrub layer 2 covered a large extent of the middle islets (but not Nake, Long, and South islets), whereas these middle islets were covered with beach scrub layer 1 and sand in December 2021. For South islet, in 2009 there was a combination of each of the land-cover categories with a majority of open-canopy, and few pixels covered with closed-canopy. In March 2021, South islet was dominated by open-canopy and closed-canopy roughly equally, then in December 2021, closed-canopy was virtually absent, and beach scrub layer 2 was interspersed throughout the areas with open-canopy. NDVI values showed no significant differences between March 2009, March 2021, and December 2021 for Millennium Atoll; however, there were different area coverages of the values across years, hence the differences in classification can be explained by the differences in the pixels at specific locations (S3 Fig).

**Fig 8:**
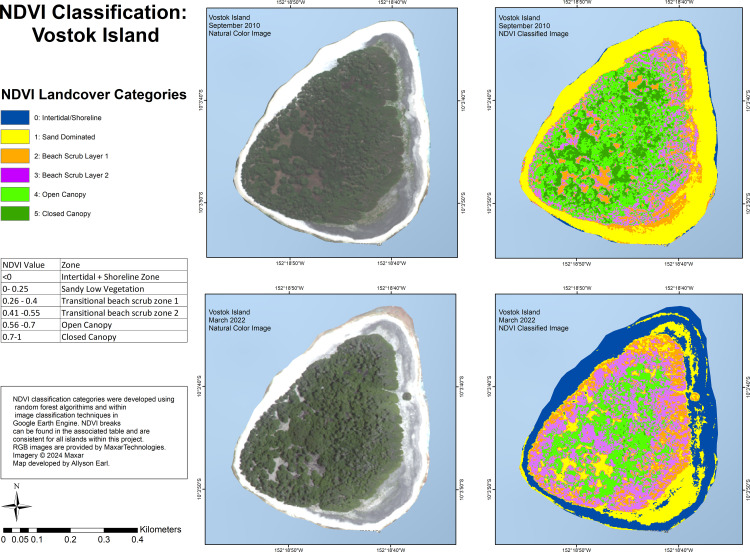
Land-cover classification using NDVI values for Vostok Island for imagery from September 2010 and March 2022.

For Vostok Island (Fig 8), in 2010 the island was dominated with open-canopy throughout the centre of the island, interspersed with closed-canopy, and beach scrub 1 and 2 extended around the perimeter, with some areas extending into the interior of the island. In 2022, intertidal + shoreline covered most of the perimeter (which in 2009 had been dominated by sand), beach scrub layer 2 showed a greater extent than in 2010, open-canopy was diminished, and closed-canopy was absent.

## Discussion and conclusions

We characterized the current vegetation of the Southern Line Islands, including Flint Island, Millennium Atoll, and Vostok Island. By comparing our results to historical studies, we found that species richness has shifted over time with succession; we saw a reduction, and in some cases disappearance, of non-native species on the islands, and an increase in native species. Flint, Millennium, and Vostok had significantly different species compositions, but we saw few differences in species frequencies, cover, beta-diversity and species turnover within the islands between 2009 and 2021. Our remote sensing results indicate some influence of dry conditions on vegetation following the ENSO event of 2015–2016.

### Progression of succession since historic studies

The trends in the plant species documented in the historic studies compared to the present study were different for each island, and our study updates the extant botanical richness of the islands. For Flint Island, we found essentially the same native species as in the Fosberg and St. John study from 1937, as well as locating a few additional native species not found in the historic study, including *Hibiscus tiliaceus* and *Terminalia catappa.* The seeds from these species are water dispersed. The differences between our observation and the historic study by St. John and Fosberg lies in the reduction of introduced species that we found. In the past, such species were under cultivation by the people running the coconut plantations, which were still active when St. John and Fosberg visited the island. The coconut plantations were abandoned several decades ago (exact date unknown). The reduction in the introduced species following the abandonment of the coconut plantations is positive for native species to experience somewhat reduced competition for resources, yet the remaining predominance of *Cocos nucifera* is of concern as it is inhibiting natural regeneration of native species in the large area still covered by *Cocos*.

For Millennium Atoll, there were a few differences between the botanical inventories in the historical studies compared to our study of importance to note. From the documentation by Bennett [70] or Dixon and Trelease [28,29] in the 19^th^ century, several species were identified that we did not find, such as *Ipomea batatas*, *Ficus carica*, and *Cucurbita pepo,* which were introduced in the era of guano extraction and when the coconut plantations were active*.* The species we documented were largely the same as those documented by Clapp and Sibley (1971a) and Kepler & Kepler (1994), while a few species such as *Laportea ruderalis* were found in these prior studies but not in ours, or vice versa, such as *Colubrina asiatica.* Additionally, in the Kepler & Kepler (1994) study, already three decades ago, it was noted that many of the islets had recovered to composition of entirely native flora following the past disturbances for economic production, yet other islets were in various stages of ecological succession following the disturbances.

Differences between our study and the historic studies were limited for Vostok Island, due to the diminutive size of the island and the low species richness (only four plant species are present currently). In the past studies from 1937 and 1971, only *Boerhavia repens* (erroneously labelled as *B. diffusa* in 1937) and *Pisonia grandis* were documented, whereas we documented, additionally, *Heliotropium arboreum* and *Euphorbia* sp*.* In the ICBP expedition, only *Boerhavia repens*, *Boerhavia tetrandra*, and *Pisonia grandis* were found. Given that the *Heliotropium arboreum* individual was small in 2009, it is likely that it dispersed to the island well after the 1937 study, and probably after the 1990 ICBP expedition was conducted, indicating why it was not found in the previous studies.

Studies on succession on other islands and atolls have shown variable results. For example, consistent with our findings, in the Solomon Islands, after abandonment of crop cultivation, non-native species disappear or experience reductions in frequency [5,71]. In contrast, in Palmyra Atoll and The Great Barrier Reef in Australia, non-native species have not disappeared following human disturbance [72]. Palmyra Atoll was studied in 1958 following several decades of industrial-military disturbance, revealing that ornamental non-native plants were introduced, then later when commercial activities were abandoned, there was a proliferation of the non-native species present and reduction of cover by natives [73]. Palmyra Atoll experiences approximately double the annual rainfall compared to the Southern Line Islands, which likely contributed to the proliferation of non-native species on the former [74]. More recently on Palmyra, ecosystem restoration efforts have begun to remove non-native species including *Cocos nucifera* and ornamental species, and to plant mixed-species native forests; this is projected to enhance carbon storage throughout the atoll [75]. In our study, we did not see the proliferation of non-native plant species, but we do see the continued prevalence of some non-native species (e.g., *Cocos nucifera*). Coconut plantations have been shown to maintain monodominance on reef islands due to their large seed size, persistence in the seed bank, and lack of limitation by propagules [76]. Similar restoration interventions to Palmyra Atoll could be explored in the Southern Line Islands, which we discuss further in our recommendations below.

### Differences across islands due to land-use history

The differences in species composition across the islands visualized in the NMDS plot can be explained by the differences in land-use histories and geographies of the islands. Vostok Island is by far the smallest, which can explain why it has the lowest species richness, and has little to no evidence of past land-use, indicating why it remains dominated by native *Pisonia* forest. Furthermore, over time on Pacific reef islands and atolls, native broadleaf forests progress into monodominance by *Pisonia,* due to ectomycorrhizal fungus associations with seabird guano, thus experiencing reductions in native plant species diversity [45,77,78]. This trend aligns with the current successional stage we see on Vostok. Flint Island has a different species composition from Vostok or Millennium, as the interior of the island remains dominated by *Cocos nucifera* following the history of the coconut plantations. Millennium Atoll has a diversity of small islets, and three large islets, allowing for species migrations across the islets, and a larger area overall to host more species. Additionally, the history of coconut plantations for copra production, as well as guano extraction throughout the atoll has also altered the plant species composition, as we still see the presence of non-native species on some islets. Across Flint, Vostok, and Millennium, the finding that beta-diversity was moderate, turnover was low, and nestedness was low accords with the significant differences in species composition across the islands and atoll. In a recent review of atolls in the Indo-Pacific and Caribbean basin, vascular plant species turnover was higher between atolls than within atolls (comparing islets), and beta-diversity within atolls was high; turnover increased with increasing cyclone frequency [79]. Our beta-diversity and turnover values tended to be lower than in the review, which may be related to low cyclone frequency.

The minor differences we observed in the field data between 2009 and 2021 may be explained by sampling, and potentially by plant responses to variation in climate. The differences in species frequencies across the years on Flint Island and Millennium Atoll were likely due to sampling. For example, on Millennium, *Hernandia nymphaeaefolia* was found in 2009 but not 2021; this species was rare (only one individual), hence suggesting why it was not found in the second field survey. Differences in species cover on Flint Island and Millennium Atoll may have resulted from different plant species responses to the ENSO event, responses to desiccation, or implications of succession post-disturbance. On Flint Island, *Cocos nucifera* and *Heliotropium arboreum* decreased in cover*,* and on Millennium, *Achyranthes aspera* and *Heliotropium arboreum* decreased*. Heliotropium arboreum* has been shown to be a salt-tolerant and drought-resistant species [80], yet showed declines in cover nevertheless. There may be novel species interactions or loss of resilience of the individuals [81] occurring on Flint Island and Millennium Atoll, due to the substantial changes to the ecosystem resulting from the disturbance by the coconut plantations (on Flint) and guano harvesting (on Millennium), or the decline may be due to species displacement by secondary broadleaf species [82]. Furthermore, on Millennium, *Cordia subcordata, Heliotropium anamolum, Pisonia grandis*, and *Portulaca lutea* increased in cover, suggesting that these species may be responding more favourably to the processes of natural succession, or better adapted to the varying climatic conditions. On Vostok, both the surveys and the NMDS indicate no detected change in plant composition. We did, however, observe growth of the single *Heliotropium arboreum* individual from 2009 to 2021, which—despite the drying conditions on the island—grew substantially over the 11 years, consistent with it being a drought-resistant species.

### Evidence of dry conditions following ENSO

The differences we found in NDVI values, and land-cover classification based on the NDVI values between 2009 (2010 for Vostok) and 2021 (2022 for Vostok) and our field observations, support our hypotheses that there is some evidence of dry conditions on the islands due to the 2015 ENSO event, and this differs across the islands. For Flint, we saw a reduction in cover by closed-canopy vegetation between 2009 and 2021, which corresponds with the reduction in NDVI values by 2021.

On Millennium, the remote sensing analysis did not show evidence of dry conditions post-ENSO in 2021 compared to Pre-ENSO 2009, but the NDVI classifications showed idiosyncrasies. In 2009, we saw a mix of the land-cover categories across the islets; then in 2021, the main change was the predominance of beach scrub layer 2 in March, versus beach scrub layer 1 and sand in December. Despite there being higher precipitation in December in the Southern Line Islands than in March, the reduction in NDVI values corresponding with the shift from beach scrub layer 2 to beach scrub layer 1 could relate to a particularly dry summer in 2021 (see S2 Fig), therefore the vegetation was not yet replenished with precipitation entering into the winter season. There may also have been shifting sand levels into the winter, as the levels of sand in December were high across the islets compared to March. Based on our field observations of Millennium, *Pisonia grandis* were showing signs of drought stress in 2021 compared to 2009. Furthermore, on South Islet, the *Cocos nucifera* were stressed with yellow fronds, and appeared to be dying out by 2021, making space for the regeneration of *Pisonia* individuals. This also allowed for the predominance of weedy understory species such as *Ipomoea* sp. Additionally, throughout the islets of Millenium Atoll, the stands of *Cordia subcordata* were showing some evidence of stress in 2021 compared to 2009 but were still considered robust.

Lastly, on Vostok, we saw evidence of dry conditions post-ENSO. There was a lack of closed-canopy cover in 2022 compared to 2010, a reduction in open-canopy, and expansion of beach scrub layer 1 and 2 compared to the denser green vegetation categories. In our field observations in 2021 on Vostok, the *Pisonia grandis* trees were less dense and less vigorous, and showed signs of stress, including dead tops.

Our land cover classification is comparable in some ways and distinct in others to a recent study by Burnett et al. (2024) classifying coconut plantations across Pacific Islands [83]. That study used four categories: coconut canopy and broadleaf tree canopy (both comparable to our open canopy and closed canopy categories), low vegetation (comparable to our beach scrub 1 and 2 categories), and non-vegetated surfaces (comparable to our intertidal/shoreline and sand dominated categories) [83]. Our classification includes six land-use categories, and was created based on NDVI values and knowledge of species present on the ground from our field surveys. From the study’s open-access data, Flint Island was shown to be covered by 53.65% coconut canopy, which accords with our findings, while Vostok was shown to be covered by 2.77% coconut canopy, which is not the case from our field surveys, and indicates the importance of using vegetation surveys on-the-ground in combination with remote sensing land use classifications.

Overall, our remote sensing analysis combined with field observations provided evidence of dry conditions in the Southern Line Islands. To date, few studies have presented findings on the impacts of the 2015–2016 ENSO event on vegetation communities in the Pacific. One study that used remote sensing land-cover classifications in Fiji found a reduction in vegetation cover with the recent ENSO event, followed by a rebound in cover since then [84]. Recent research has shown variable changes in Leaf Area Index after ENSO events for islands in the Central-Pacific versus Eastern-Pacific [85], hence such impacts appear region-specific. Additional studies on other Pacific islands would be beneficial to examine the impacts of the recent event, as well as future severe ENSO or other climatic events on small islands.

Limitations to our study included the differences in the sampling seasons from 2009 to 2021, and the time lapsed between the ENSO event and the second sampling period. Even though our test of the effect of seasons on NDVI showed no significant differences (S3 Fig), and rainfall is similar during fall and spring (S2 Fig), the fact that our surveys and satellite images were taken at different seasons may confound our results. Furthermore, given that the second time period of sampling was five years following the ENSO (six years for the satellite imagery on Vostok), other factors not studied here could also have contributed to the land use changes that we saw. For example, in the coastal areas, changes from the beach scrub land cover to the land cover types with lower NDVI values could have been due to natural island dynamism, such as changes to coastal sediment [10]. This type of dynamism is not likely to impact the interior of the islands, however, where climatic changes are the most probable drivers of change; we also noted the trend in drying and stress for the *Pisonia*, and *Cocos* in the island interiors from our field observations**.**

### Recommendations for ecosystem restoration and future research

Based on our results, we recommend that active ecological restoration interventions would be beneficial for the terrestrial ecosystems of the Southern Line Islands. On Flint, the predominance of *Cocos nucifera* remaining decades after the abandonment of the plantations could warrant the removal of coconut palms and planting of *Pisonia grandis*. On Millennium Atoll, removal of *Cocos nucifera* may be unnecessary as individuals appear to be dying naturally due to the changing climatic conditions. Few studies exist on methodologies for restoring native forests on small islands after abandonment of coconut plantations. One study in the Seychelles showed that removing all *Cocos nucifera* individuals other than canopy individuals, to retain shade as the native trees and shrubs are growing, following by successive thinning of mature *Cocos*, is more time and cost effective than clear cutting [50]. Clear-cutting had been undertaken in other islands in the Seychelles, with plantings of native trees to restore habitat for the Seychelles warbler (*Acrocephalus sechellensis*) [86,87]. Such an approach of selective cutting and successive thinning could be tested on Flint, or a comparison of methods of removal of the coconut palms and restoration of the native *Pisonia grandis* forest, including supplemental plantings of other native species, could be tested.

Furthermore, on Vostok the presence of non-native Polynesian rats (*Rattus exulans*) is currently the main hindrance to the health of the rare, native *Pisonia* forest. In our field surveys in 2021, we found that there was a population of at least one thousand. Rats are present on each of Flint, Vostok, and Millennium. Hence, the management of their populations could be advantageous to the native species of each island, particularly Vostok which could benefit at the lowest cost given the small size of the island. A worldwide review of invasive rodent removal has shown variable success using different methods [88], but overall this intervention has been recommended to prevent extirpation of native animal species, particularly native seabirds, and to support the long-term health of native vegetation [89].

Future research could include similar studies of ecological succession on other small islands and atolls in the Pacific with histories of disturbance due to commercial land-use, as well as the testing of ecosystem restoration approaches. Studies testing the efficacy of methods for the restoration of native ecosystems on other small islands, such as mentioned above for Flint Island, would be beneficial. In the Southern Line Islands, further studies could be conducted in collaboration with local and Indigenous knowledge holders from Kiribati, to include local views and values in the intended objectives and design of ecological restoration and conservation efforts.

## Supporting information

S1 Table NDVI breaks and land-cover category descriptions for the Southern Line Islands. (PDF)

S2 Table NDVI classification metrics across Flint Island, Millennium Atoll and Vostok Island. (PDF)

S3 Table Frequency of plant species on Flint Island. Data shown for 2009 and 2021, as the number of plots in which the species were found in areas that were surveyed in both years. Dashes indicate the species was not found.
(PDF)

S4 Table Frequency of plant species on Millennium Atoll. Data shown for 2009 and 2021, as the number of plots in which the species were found in areas that were surveyed in both years. Dashes indicate the species was not found.
(PDF)

S5 Table Frequency of plant species on Vostok Island. Data shown for 2009 and 2021, as the number of plots in which the species were found in areas that were surveyed in both years.
(PDF)

S6 Table Average percent cover of plant species on Flint Island. Comparisons shown for 2009 versus 2021, for locations where plots were conducted in both years. Calculations for species with multiple individuals recorded and estimated in both 2009 and 2021 using Wilcoxon rank sum test (unpaired data; function wilcox.test in R). Significant p-values denoted with an asterisk. For species with Standard Deviation (SD) listed as NA, only one individual was found. Dashes indicate the species was not found.
(PDF)

S7 Table Average percent cover of plant species on Millennium Atoll. Comparisons shown for 2009 versus 2021, for locations where plots were conducted in both years. Calculations for species with multiple individuals recorded and estimated in both 2009 and 2021 using Wilcoxon rank sum test (unpaired data; function wilcox.test in R). Significant p-values denoted with an asterisk. For species with Standard Deviation (SD) listed as NA, only one individual was found. Dashes indicate the species was not found.
(PDF)

S8 Table Average percent cover of plant species on Vostok Island. Comparisons shown for 2009 versus 2021, for locations where plots were conducted in both years. Calculations for species with multiple individuals recorded and estimated in both 2009 and 2021 using Wilcoxon rank sum test (unpaired data; function wilcox.test in R). Significant p-values denoted with an asterisk. For species with Standard Deviation (SD) as NA, only one individual was found.
(PDF)

S9 Table Beta-diversity, species turnover and nestedness analysis for the flora of the Southern Line Islands. Results of beta-diversity Sørensen dissimilarity index (beta.sor in R), species turnover Simpson dissimilarity index (beta.sim in R), and nestedness (beta.sne in R), within island for 2009 versus 2021, and across islands within years, for Flint Island, Millennium Atoll, and Vostok Island. The calculation for beta.sor = beta.sim + beta.sne.
(PDF)

S1 Fig Species accumulation curves for the flora of the Southern Line Islands. Curves constructed for Flint Island in 2009 (A), Flint Island in 2021 (B), Millennium Atoll in 2009 (C), and Millennium Atoll in 2021 (D), using the package vegan in R, for all vegetation plots conducted in the field. Curves not constructed for Vostok Island due to the small number of plots and species richness.
(PDF)

S2 Fig Mean monthly precipitation for the Southern Line Islands. Values shown for Flint Island (A), Millennium Atoll (B), and Vostok Island (C) from 2008–2022. Data extracted from CHIRPS: Rainfall Estimates from Rain Gauge and Satellite Observations, from the Climate Hazards Center at UC Santa Barbara [58]. Note that data from the database was not available for January or December 2017 for any location. Annual precipitation trends are similar across the islands. Seasonality in the Southern Line Islands is observed, with the greatest precipitation in the winter months from November to February, then a decline from March to May, followed by the lowest precipitation in the summer months from May to August, then an increase through September and October. Considering that the field surveys were conducted in April 2009 and October 2021, precipitation levels are similar at these times of the year, albeit slightly higher in October than April. Seasonal differences in precipitation are not expected to substantially impact the vegetation characteristics from the periods of the year in which the field surveys were conducted, whereas differences across the entire year would inform the health and vigor of the vegetation at a given time. In the ENSO years of 2015 and 2016, for all islands, for most months we saw slight reductions in precipitation compared to years prior and after. From post ENSO to 2022, there were stochastic increases and decreases in precipitation. Corresponding with the remote sensing images, for Flint Island, March 2009 and September 2021 showed precipitation of 186.35 mm and 134.05 mm respectively. For Millennium Atoll, March 2009, March 2021, and December 2021 showed monthly mean precipitation values of 203.74 mm, 199.76 mm, and 284.18 mm respectively. Lastly, for monthly mean precipitation on Vostok Island, September 2010 and March 2022 showed values of 130.11 mm and 203.83 mm respectively, when the available remote sensing images were selected.
(PDF)

S3 Fig NDVI values for Millennium Atoll. Boxplots (A) and density plots (B) showing the spread of NDVI values for Millennium Atoll for March 2009, March 2021 and December 2021.
(PDF)
