# Supplementary material for: Influence of land-use history and ENSO on the flora of the Southern Line Islands
Source: PLoS One. 2026 Feb 6;21(2):e0341582. doi: 10.1371/journal.pone.0341582 (PMC12880752; doi:10.1371/journal.pone.0341582)
Supplement: S1 Table — (PDF) [file pone.0341582.s001.pdf]

S1 Table. NDVI breaks and land-cover category descriptions for the Southern Line Islands.

Range of NDVI values and zone names	Description of zones
<0 **Intertidal zone + shoreline**	The intertidal zone + shoreline contains a mixture of land-cover types. Due to the ephemeral nature of the shoreline in small islands and atolls, and the sensitivity of NDVI to soil color, this zone was developed to distinguish darker rock and algae-covered regions from areas further up the shoreline containing beach plants and wet soil. Additionally, this region can be covered in water which has a negative NDVI value in most cases, and rather than masking out these values which would cause differences in shoreline between images, this category allowed for a consistent image footprint.
0 - 0.25 **Sand dominated**	This zone was developed to capture bright, light-colored sand. Generally low in vegetation, this region will have variable spectral signatures due to how wet or dry the sand is. For the purposes of this project, wet and dry sand categories were lumped together as distinguishing between these two states was unnecessary.
0.26 - 0.4 **Transitional beach scrub zone 1**	Transitions between sandy shoreline and the beginnings of vegetation cover were challenging to distinguish using NDVI, so multiple transitional categories were developed to capture the complexity of the beach front vegetation and the changing reflectance of sand. Beach scrub layer 1 captures the start of shoreline vegetation, senescing grasses or other vegetation that may be present, and would be missed by the naked eye.
0.41 - 0.55 **Transitional beach scrub zone 2**	Beach scrub layer 2 was developed to capture established beach vegetation that may be present in sandy gaps in the interior of the island, and transitional regions where vegetation coverage generally increases towards the interior of the island. Despite being a small zone to capture, it adds context to the unique variability present in the Southern Line Islands.
0.56 - 0.7 **Open-canopy**	The open-canopy zone was developed for values 0.56-0.7, and while these values can be found within closed-canopy regions, they are highly dependent on seasonality. This zone was deemed necessary, given the general trend in the high-resolution imagery used in our study to capture the spatial nuance of the islands and atoll, and the changing density of foliage.
0.7 - 1 **Closed-canopy**	Closed-canopy zones were defined as the highest NDVI values. High NDVI values may be found throughout the images and not exclusively in closed-canopy zones. However, the density of these values was generally found in the interior of the atolls where it can be assumed that vegetation is established and dense.
