# Supplementary material for: Influence of land-use history and ENSO on the flora of the Southern Line Islands
Source: PLoS One. 2026 Feb 6;21(2):e0341582. doi: 10.1371/journal.pone.0341582 (PMC12880752; doi:10.1371/journal.pone.0341582)
Supplement: S2 Table — (PDF) [file pone.0341582.s002.pdf]

S2 Table. NDVI classification metrics across Flint Island, Millennium Atoll and Vostok Island.

	Flint 2009	Flint 2021	Millennium March 2009	Millennium March 2021	Millennium December 2021	Vostok 2010	Vostok 2022
**Training Accuracy**	98.75%	99.46%	99.90%	99.94%	99.93%	99.40%	99.78%
**Validation Accuracy**	87.44%	85.44%	99.54%	99.86%	99.76%	99.13%	99.51%
**Kappa Test**	84.92%	82.52%	99.44%	99.48%	99.71%	98.96%	99.68%
