# Supplementary material for: Influence of land-use history and ENSO on the flora of the Southern Line Islands
Source: PLoS One. 2026 Feb 6;21(2):e0341582. doi: 10.1371/journal.pone.0341582 (PMC12880752; doi:10.1371/journal.pone.0341582)
Supplement: S4 Table — Data shown for 2009 and 2021, as the number of plots in which the species were found in areas that were surveyed in both years. Dashes indicate the species was not found. (PDF) [file pone.0341582.s004.pdf]

S4 Table. Frequency of plant species on Millennium Atoll. Data shown for 2009 and 2021, as the number of plots in which the species were found in areas that were surveyed in both years. Dashes indicate the species was not found.

Species	Frequency in 2009<br>Number of plots=167	Frequency in 2021<br>Number of plots=179
Achyranthes aspera	30	13
Boerhavia repens	66	57
Cocos nucifera	31	30
Colubrina asiatica	1	1
Cordia subcordata	37	58
Heliotropium anamolum	6	8
Heliotropium arboreum	79	82
Hernandia nymphaeaefolia	1	-
Ipomoea sp.	14	3
Laportea aestuans	17	2
Lepturus repens	18	16
Microsorum scolopendria	28	25
Morinda citrifolia	47	26
Pandanus sp.	4	5
Pisonia grandis	71	82
Portulaca lutea	45	41
Suriana maritima	2	3
