# Supplementary material for: Influence of land-use history and ENSO on the flora of the Southern Line Islands
Source: PLoS One. 2026 Feb 6;21(2):e0341582. doi: 10.1371/journal.pone.0341582 (PMC12880752; doi:10.1371/journal.pone.0341582)
Supplement: S5 Table — Data shown for 2009 and 2021, as the number of plots in which the species were found in areas that were surveyed in both years. (PDF) [file pone.0341582.s005.pdf]

S5 Table. Frequency of plant species on Vostok Island. Data shown for 2009 and 2021, as the number of plots in which the species were found in areas that were surveyed in both years.

Species	Frequency in 2009<br>Number of plots=10	Frequency in 2021<br>Number of plots=8
Boerhavia repens	2	1
Euphorbia sp.	2	1
Pisonia grandis	8	7
