# Supplementary material for: Influence of land-use history and ENSO on the flora of the Southern Line Islands
Source: PLoS One. 2026 Feb 6;21(2):e0341582. doi: 10.1371/journal.pone.0341582 (PMC12880752; doi:10.1371/journal.pone.0341582)
Supplement: S7 Table — Comparisons shown for 2009 versus 2021, for locations where plots were conducted in both years. Calculations for species with multiple individuals recorded and estimated in both 2009 and 2021 using Wilcoxon rank sum test (unpaired data; function wilcox.test in R). Significant p-values denoted with an asterisk. For species with Standard Deviation (SD) listed as NA, only one individual was found. Dashes indicate the species was not found. (PDF) [file pone.0341582.s007.pdf]

S7 Table. Average percent cover of plant species on Millennium Atoll. Comparisons shown for 2009 versus 2021, for locations where plots were conducted in both years. Calculations for species with multiple individuals recorded and estimated in both 2009 and 2021 using Wilcoxon rank sum test (unpaired data; function wilcox.test in R). Significant p-values denoted with an asterisk. For species with Standard Deviation (SD) listed as NA, only one individual was found. Dashes indicate the species was not found.

Species	2009 Average Percent Cover (SD)	2021 Average Percent Cover (SD)	Test Statistic (W)	p-value
Achyranthes aspera	24.43 (18.07)	9.23 (8.79)	301	0.0047*
Boerhavia repens	35.03 (18.30)	32.23 (24.04)	2075	0.32
Cocos nucifera	40.35 (30.56)	32.57 (30.77)	525.5	0.38
Colubrina asiatica	80.00 (NA)	70.00 (NA)	-	-
Cordia subcordata	41.49 (24.88)	58.28 (31.01)	730	0.0086*
Heliotropium anamolum	7.83 (7.05)	28.13 (11.93)	3.5	0.0090*
Heliotropium arboreum	51.34 (20.93)	38.13 (18.88)	4496.5	1.77e-05*
Ipomoea sp.	13.33 (7.64)	49.79 (32.14)	33.5	0.13
Laportea aestuans	14.18 (13.62)	1.50 (0.71)	27.5	0.18
Lepturus repens	9.39 (9.13)	13.00 (8.47)	103	0.16
Microsorum scolopendria	30.00 (16.72)	27.48 (18.31)	383.5	0.55
Morinda citrifolia	22.09 (15.42)	24.19 (15.83)	559	0.55
Pandanus sp.	47.75 (45.39)	38.40 (47.12)	11	0.90
Pisonia grandis	42.08 (24.95)	47.74 (28.09)	2588	0.24
Portulaca lutea	22.02 (13.94)	34.80 (20.89)	567	0.0019*
Suriana maritima	15.00 (7.07)	36.67 (15.28)	0.5	0.24
