# Supplementary material for: Influence of land-use history and ENSO on the flora of the Southern Line Islands
Source: PLoS One. 2026 Feb 6;21(2):e0341582. doi: 10.1371/journal.pone.0341582 (PMC12880752; doi:10.1371/journal.pone.0341582)
Supplement: S8 Table — Comparisons shown for 2009 versus 2021, for locations where plots were conducted in both years. Calculations for species with multiple individuals recorded and estimated in both 2009 and 2021 using Wilcoxon rank sum test (unpaired data; function wilcox.test in R). Significant p-values denoted with an asterisk. For species with Standard Deviation (SD) as NA, only one individual was found. (PDF) [file pone.0341582.s008.pdf]

S8 Table. Average percent cover of plant species on Vostok Island. Comparisons for 2009 versus 2021, for locations where plots were conducted in both years. Calculations for species with multiple individuals recorded and estimated in both 2009 and 2021 using Wilcoxon rank sum test (unpaired data; function wilcox.test in R). Significant p-values denoted with an asterisk. For species with Standard Deviation (SD) as NA, only one individual was found.

Species	2009 Average Percent Cover (SD)	2021 Average Percent Cover (SD)	Test Statistic (W)	p-value
Boerhavia repens	30.00 (28.28)	30.00 (NA)	1	1.0
Euphorbia sp.	75.00 (35.36)	35.00 (NA)	2	0.66
Pisonia grandis	87.88 (34.29)	97.14 (7.56)	28	1.0
