# Supplementary material for: Influence of land-use history and ENSO on the flora of the Southern Line Islands
Source: PLoS One. 2026 Feb 6;21(2):e0341582. doi: 10.1371/journal.pone.0341582 (PMC12880752; doi:10.1371/journal.pone.0341582)
Supplement: S9 Table — Results of beta-diversity Sørensen dissimilarity index (beta.sor in R), species turnover Simpson dissimilarity index (beta.sim in R), and nestedness (beta.sne in R), within island for 2009 versus 2021, and across islands within years, for Flint Island, Millennium Atoll, and Vostok Island. The calculation for beta.sor = beta.sim + beta.sne. (PDF) [file pone.0341582.s009.pdf]

S9 Table. Beta-diversity, species turnover and nestedness analysis for the flora of the Southern Line Islands. Results of beta-diversity Sørensen dissimilarity index (beta.sor in R), species turnover Simpson dissimilarity index (beta.sim in R), and nestedness (beta.sne in R), within island for 2009 versus 2021, and across islands within years, shown for Flint Island, Millennium Atoll, and Vostok Island. The calculation for beta.sor = beta.sim + beta.sne.

**Island Comparisons**	**Beta-diversity, Sørensen dissimilarity index (beta.sor in R)**	**Species turnover, Simpson dissimilarity index (beta.sim in R)**	**Nestedness (beta.sne in R)**
Flint 2009 - Flint 2021	0.294	0	0.294
Millennium 2009 - Millennium 2021	0	0	0
Vostok 2009 - Vostok 2021	0	0	0
Flint 2009 - Millennium 2009	0.455	0	0.455
Flint 2021 - Millennium 2021	0.333	0.182	0.152
Millennium 2009 - Vostok 2009	0.789	0.333	0.456
Millennium 2021 - Vostok 2021	0.789	0.333	0.456
Vostok 2009 - Flint 2009	0.714	0.667	0.111
Vostok 2021 - Flint 2021	0.778	0.333	0.381
Vostok - Millennium - Flint 2009	0.667	0.273	0.394
Vostok - Millennium - Flint 2021	0.607	0.267	0.340
