# Supplementary material for: Influence of land-use history and ENSO on the flora of the Southern Line Islands
Source: PLoS One. 2026 Feb 6;21(2):e0341582. doi: 10.1371/journal.pone.0341582 (PMC12880752; doi:10.1371/journal.pone.0341582)
Supplement: S1 Fig — Curves constructed for Flint Island in 2009 (A), Flint Island in 2021 (B), Millennium Atoll in 2009 (C), and Millennium Atoll in 2021 (D), using the package vegan in R, for all vegetation plots conducted in the field. Curves not constructed for Vostok Island due to the small number of plots and species richness. (PDF) [file pone.0341582.s010.pdf]

S1 Fig. Species accumulation curves for the flora of the Southern Line Islands. Curves constructed for Flint Island in 2009 (A), Flint Island in 2021 (B), Millennium Atoll in 2009 (C), and Millennium Atoll in 2021 (D), using the package vegan in R, for all vegetation plots conducted in the field. Curves not constructed for Vostok Island due to the small number of plots and low species richness.

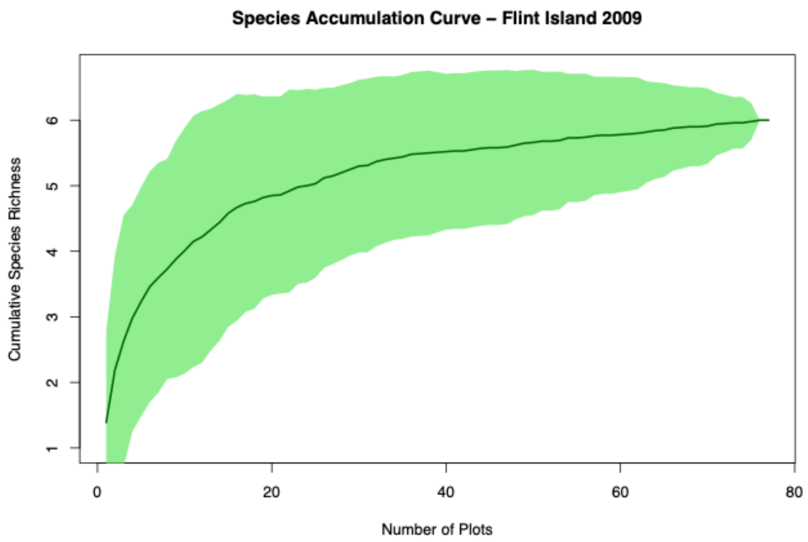

A.

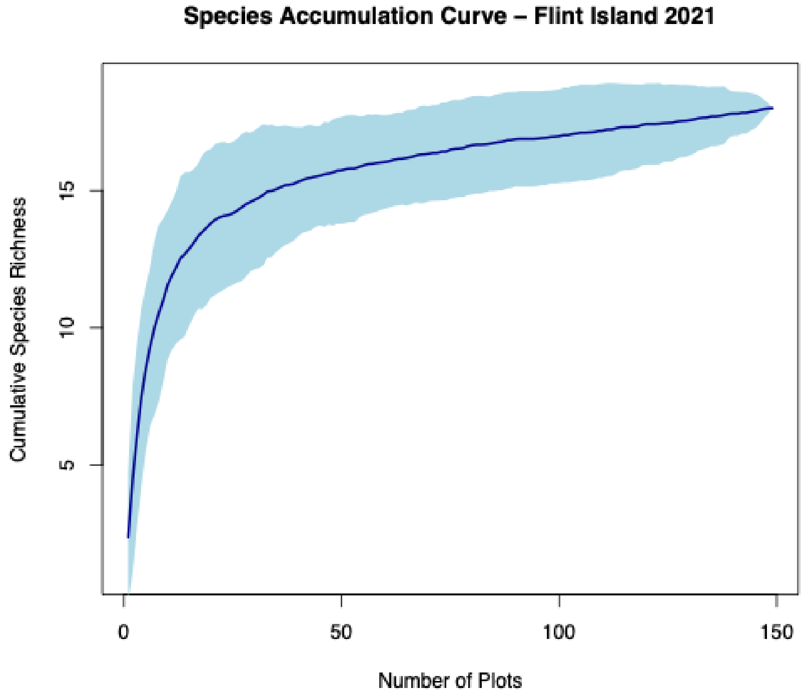

B.

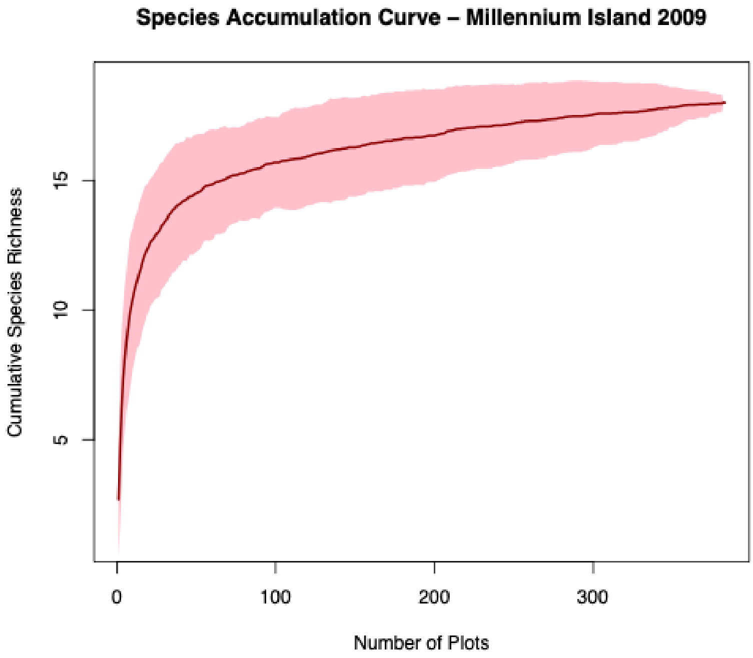
Species Accumulation Curve – Millennium Island 2009
Cumulative Species Richness
Number of Plots
5
10
15
0
100
200
300

C.

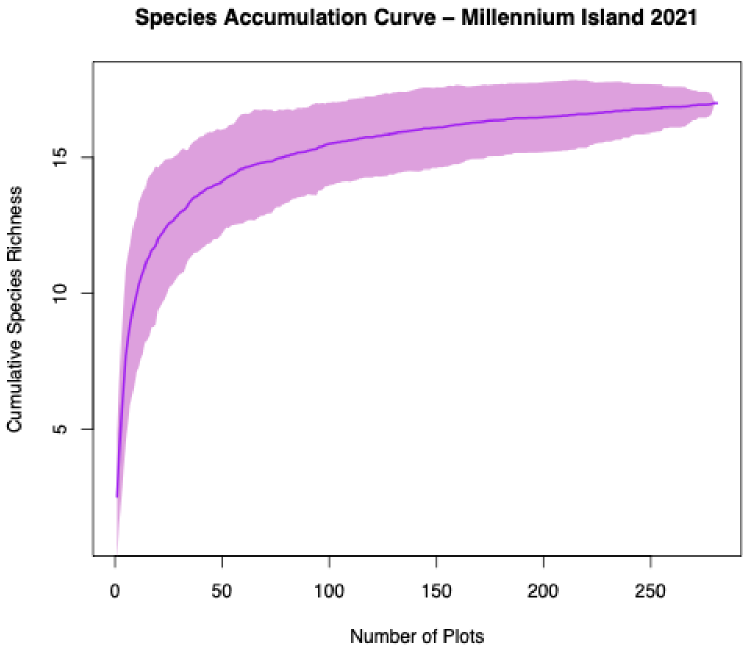
Species Accumulation Curve – Millennium Island 2021
Cumulative Species Richness
Number of Plots
5
10
15
0
50
100
150
200
250

D.
