# Supplementary material for: Influence of land-use history and ENSO on the flora of the Southern Line Islands
Source: PLoS One. 2026 Feb 6;21(2):e0341582. doi: 10.1371/journal.pone.0341582 (PMC12880752; doi:10.1371/journal.pone.0341582)
Supplement: S2 Fig — Values shown for Flint Island (A), Millennium Atoll (B), and Vostok Island (C) from 2008–2022. Data extracted from CHIRPS: Rainfall Estimates from Rain Gauge and Satellite Observations, from the Climate Hazards Center at UC Santa Barbara [58]. Note that data from the database was not available for January or December 2017 for any location. Annual precipitation trends are similar across the islands. Seasonality in the Southern Line Islands is observed, with the greatest precipitation in the winter months from November to February, then a decline from March to May, followed by the lowest precipitation in the summer months from May to August, then an increase through September and October. Considering that the field surveys were conducted in April 2009 and October 2021, precipitation levels are similar at these times of the year, albeit slightly higher in October than April. Seasonal differences in precipitation are not expected to substantially impact the vegetation characteristics from the periods of the year in which the field surveys were conducted, whereas differences across the entire year would inform the health and vigor of the vegetation at a given time. In the ENSO years of 2015 and 2016, for all islands, for most months we saw slight reductions in precipitation compared to years prior and after. From post ENSO to 2022, there were stochastic increases and decreases in precipitation. Corresponding with the remote sensing images, for Flint Island, March 2009 and September 2021 showed precipitation of 186.35 mm and 134.05 mm respectively. For Millennium Atoll, March 2009, March 2021, and December 2021 showed monthly mean precipitation values of 203.74 mm, 199.76 mm, and 284.18 mm respectively. Lastly, for monthly mean precipitation on Vostok Island, September 2010 and March 2022 showed values of 130.11 mm and 203.83 mm respectively, when the available remote sensing images were selected. (PDF) [file pone.0341582.s011.pdf]

S2 Fig. Mean monthly precipitation for the Southern Line Islands. Values shown for Flint Island (A), Millennium Atoll (B), and Vostok Island (C) from 2008-2022. Data extracted from CHIRPS: Rainfall Estimates from Rain Gauge and Satellite Observations, from the Climate Hazards Center at UC Santa Barbara [58]. Note that data from the database were not available for January or December 2017 for any location. Annual precipitation trends are similar across the islands. Seasonality in the Southern Line Islands is observed, with the greatest precipitation in the winter months from November to February, then a decline from March to May, followed by the lowest precipitation in the summer months from May to August, then an increase through September and October. Considering that the field surveys were conducted in April 2009 and October 2021, precipitation levels are similar at these times of the year, albeit slightly higher in October than April. Seasonal differences in precipitation are not expected to substantially impact the vegetation characteristics from the periods of the year in which the field surveys were conducted, whereas differences across the entire year would inform the health and vigor of the vegetation at a given time. In the ENSO years of 2015 and 2016, for all islands, for most months we saw slight reductions in precipitation compared to years prior and after. From post ENSO to 2022, there were stochastic increases and decreases in precipitation. Corresponding with the remote sensing images, for Flint Island, March 2009 and September 2021 showed precipitation of 186.35 mm and 134.05 mm respectively. For Millennium Atoll, March 2009, March 2021, and December 2021 showed monthly mean precipitation values of 203.74 mm, 199.76 mm, and 284.18 mm respectively. Lastly, for monthly mean precipitation on Vostok Island, September 2010 and March 2022 showed values of 130.11 mm and 203.83 mm respectively, when the available remote sensing images were selected.

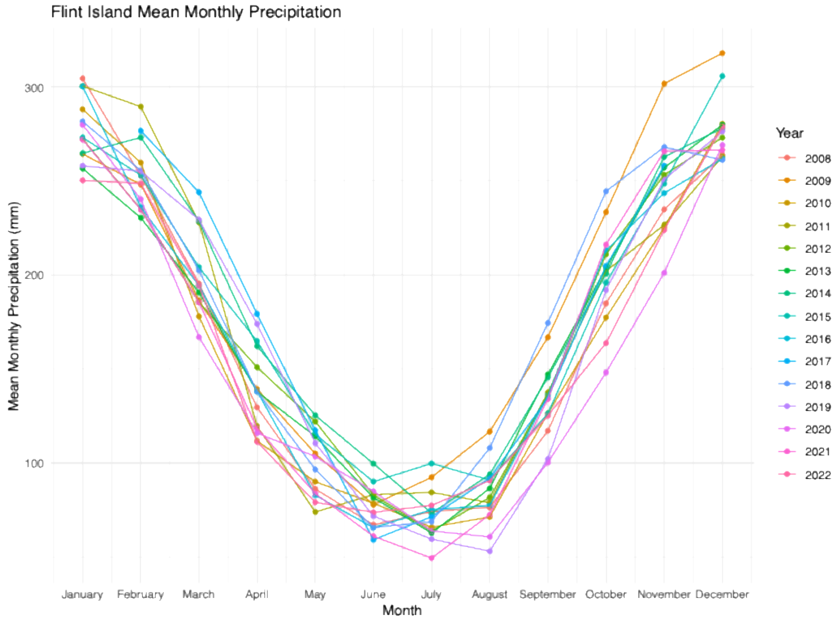

A.

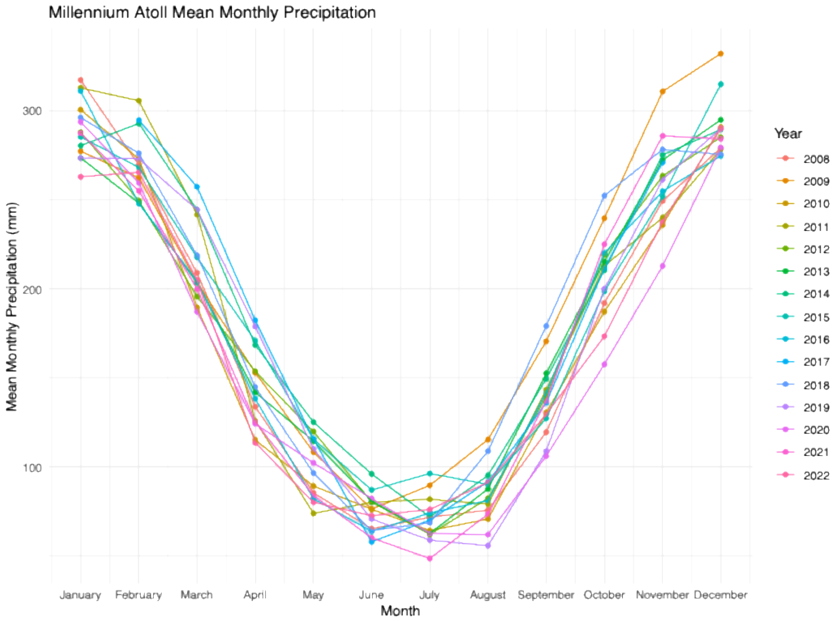

Millennium Atoll Mean Monthly Precipitation
Mean Monthly Precipitation (mm)
300
200
100
January
February
March
April
May
June
July
August
September
October
November
December
Month
Year
2008
2009
2010
2011
2012
2013
2014
2015
2016
2017
2018
2019
2020
2021
2022

B.

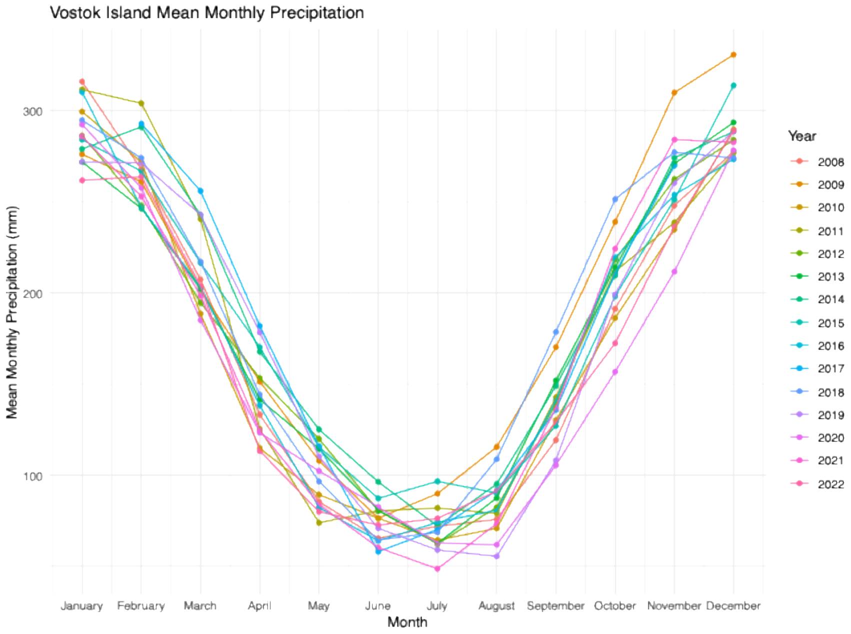

Vostok Island Mean Monthly Precipitation
Mean Monthly Precipitation (mm)
300
200
100
January
February
March
April
May
June
July
August
September
October
November
December
Month
Year
2008
2009
2010
2011
2012
2013
2014
2015
2016
2017
2018
2019
2020
2021
2022

C.
