# Supplementary material for: Influence of land-use history and ENSO on the flora of the Southern Line Islands
Source: PLoS One. 2026 Feb 6;21(2):e0341582. doi: 10.1371/journal.pone.0341582 (PMC12880752; doi:10.1371/journal.pone.0341582)
Supplement: S3 Fig — Boxplots (A) and density plots (B) showing the spread of NDVI values for Millennium Atoll for March 2009, March 2021 and December 2021. (PDF) [file pone.0341582.s012.pdf]

S3 Fig. NDVI values for Millennium Atoll. Boxplots (A) and density plots (B) showing the spread of NDVI values for Millennium Atoll for March 2009, March 2021 and December 2021.

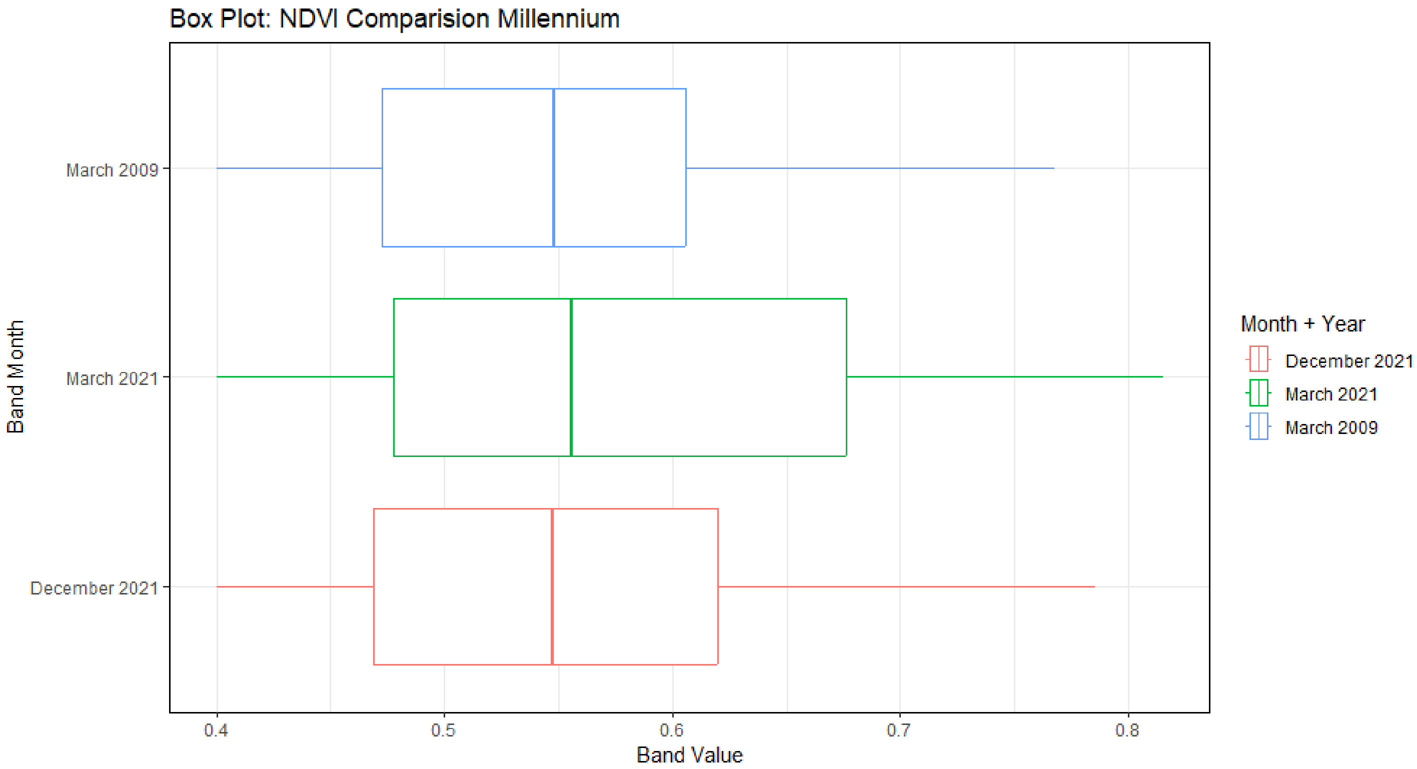

A.

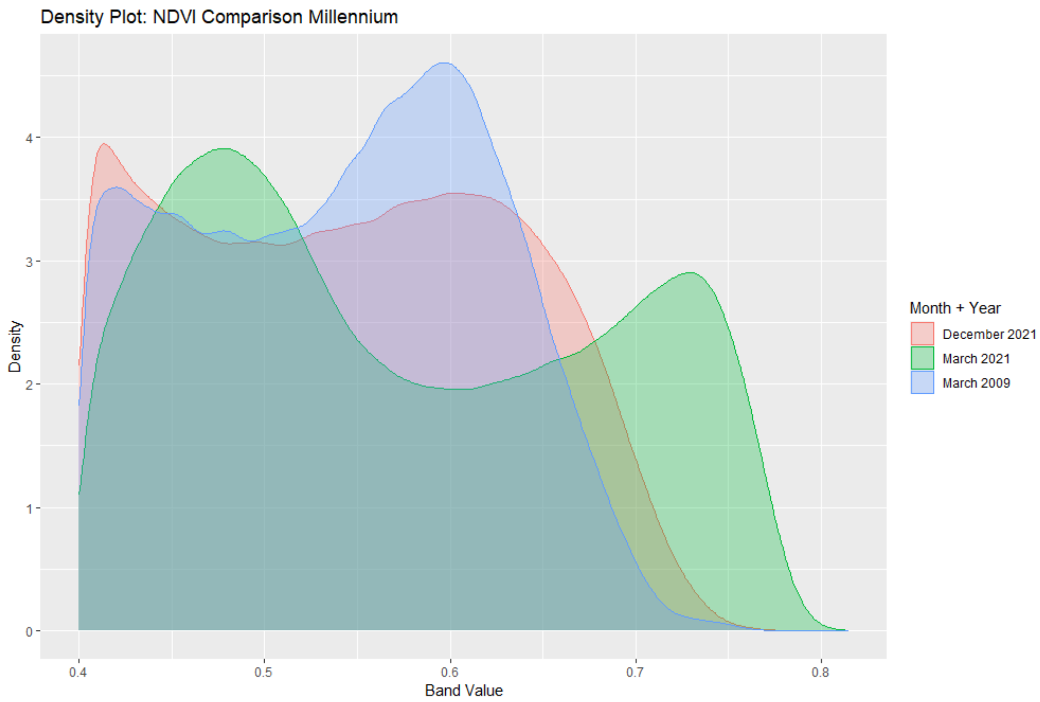

B.
